# Rapid evaporative ionisation mass spectrometry can age field caught *Anopheles gambiae* malaria vectors

**DOI:** 10.1038/s41598-025-03779-x

**Published:** 2025-06-02

**Authors:** Iris Wagner, Antoine Sanou, Moussa Guelbeogo, Jessica Williams, Joscelyn Sarsby, Ellie Sherrard Smith, Roger Sanou, Issiaka Sare, Madou Tapsoba, Zongo Soumnaba, Etienne Bilgo, Diabate Abdoulaye, Roch J. Dabire, Andrew M. Blagborough, Robert J. Beynon, Hilary Ranson

**Affiliations:** 1 Department of Vector Biology, Liverpool School of Tropical Medicine, Pembroke Place, Liverpool, L3 5QA UK; 2 Centre for Proteome Research, Institute of Systems, Molecular and Integrative Biology, University of Liverpool, Liverpool, L69 7ZB UK; 3 Centre National de Recherche et de Formation sur le Paludisme, Ouagadougou, Burkina Faso; 4 Université de Fada N’Gourma, Fada N’Gourma, Burkina Faso; 5 Université Joseph KI-ZERBO, Ouagadougou, Burkina Faso; 6 MRC Centre for Global Infectious Disease Analysis, Imperial College London, London, UK; 7 Direction Regionale de l’Ouest, Institut de Recherche en Sciences de la Santé, Bobo-Dioulasso, Burkina Faso; 8 Institut National de Santé Publique/Centre Muraz, Bobo Dioulasso, Burkina Faso; 9 Department of Pathology, University of Cambridge, Tennis Court Road, Cambridge, CB2 1QP UK

**Keywords:** Mosquito, Age-grading, Malaria, Vector control, Malaria, Mass spectrometry

## Abstract

Reliable estimates of the age structure of malaria mosquitoes would aid evaluation of the efficacy of vector control and inform models of malaria transmission. We have previously shown that rapid evaporative ionisation mass spectrometry (REIMS) could determine the age and species of laboratory reared mosquitoes and culicine mosquitoes from a river estuary in the United Kingdom. Here we investigated the robustness of this methodology by introducing additional environmental, genetic and physiological diversity in experiments using laboratory reared and field collected *Anopheles gambiae* mosquitoes. REIMS reliably separated mosquitoes into 3 or 5 age groups with > 80% accuracy using mosquitoes of mixed physiological status reared in insectaries or collected from semi-field stations in Burkina Faso. In addition, REIMS could distinguish mosquitoes that were nulliparous from those that had undergone one or two oviposition cycles with an accuracy of 87%. In a proof of principle experiment REIMS readily distinguished mosquitoes infected with *Plasmodium berghei* from non-infected. Furthermore, we show that age grading is possible using only mosquito abdomens, leaving biomass for further molecular biological experiments. Finally, we used the model generated from mixed field collections to estimate the age structure of mosquitoes collected from inside houses.

**Supplementary Information:**

The online version contains supplementary material available at 10.1038/s41598-025-03779-x.

## Introduction

Insecticide based control strategies have proven to be extremely effective in reducing the burden of vector borne disease such as malaria^1^. Interventions such as insecticide treated nets or indoor residual spraying target specific times in the vector’s gonotrophic cycle, maximising the likelihood of repeated insecticide contact and reducing the environmental impact of blanket spraying or treatment of breeding sites. These interventions have proven so successful because they reduce the daily survival rates of the adult female mosquitoes which is the key determinant of the vectorial capacity^2^; as the malaria parasite takes longer to develop in the mosquito than the expected lifespan of mosquitoes under most environmental conditions, small reductions in daily survival have a dramatic impact on the proportion of infectious mosquitoes and hence rates of transmission (reviewed in^3^).

The ability to generate reliable predictions of the age structure of natural mosquito populations would be immensely beneficial for assessing transmission risk and could be an alternative to measurements of entomological inoculation rates (EIR) which require very large sample sizes, particularly when transmission levels are low. Understanding how age structure is impacted by environmental conditions, seasonality or anthropogenic change, for example, would improve predictions from malaria transmission models. Furthermore, the combination of accurate estimations of population age structure and modelling could potentially provide a reliable indicator of the impact of vector control interventions in different ecological and epidemiological settings, reducing the need for expensive and lengthy randomised controlled trials.

Several different approaches have been applied to assess mosquito age ranging from manual dissection and counting of ovarian dilatations^4^, to molecular methods to quantify gene expression in spermatozoa^5^ to infrared spectroscopy approaches^6,7^. The advantages and limitations of the different methods have been recently reviewed^3^. Recent developments combining deep machine learning with infrared spectroscopy (DL MIRS)^8^ perhaps show the greatest promise although proponents of this method recognise that there are still several challenges to overcome before this method can be utilised as part of routine malaria surveillance programmes^9^.

We have previously reported that rapid evaporative ionisation mass spectrometry (REIMS) can reliably predict the age structure of mosquitoes in experiments using laboratory reared *Anopheles gambiae* and field caught culicine mosquitoes^10^. We showed that REIMS is a rapid approach, that can be used on dried mosquito samples and, when used with supervised machine learning, can provide data on age profiles with similar accuracy to DL MIRS. We were therefore interested in evaluating the robustness of REIMS age predictions when additional variables (physiological status, diet, temperature and humidity, exposure to xenobiotics etc.) were introduced.

In this study, after confirming that blood feeding status did not interfere with age predictions in the laboratory, we evaluated the ability of REIMS to predict age in *An. gambiae* mosquitoes from their natural habitat in southwest Burkina Faso. Encouragingly, we found that models made using mosquitoes collected from different habitats and reared under different environmental conditions (in local insectaries and outdoors in semi field stations) could be combined into a single model and then used to accurately predict age structure of mosquitoes collected the following year. We also show that REIMS can be used to detect the malaria parasite (although the current study was limited to laboratory infections). Finally, in a proof of principle experiment, we used the combined model to predict the age structure of mosquitoes collected inside homes.

The results from this study further demonstrate the applicability of REIMS as an additional method worthy of consideration for estimating mosquito age structure. Adapting this method to utilise lower cost instrumentation would potentially provide a high throughput, near reagent-free, approach for profiling mosquito populations and assessing the impact of alternative control strategies.

## Results

### The physiological status of mosquitoes does not adversely affect age grading

In previous experiments we established that REIMS signatures could be used to distinguish mosquitoes of different age groups, using laboratory strains as well as mosquitoes collected from the wild in the Dee Estuary in Northwest England^10^. However, several factors, which are of importance when identifying wild-caught mosquitoes had not been explored, such as blood-feeding and aging in natural environments.

**Fig. 1:**
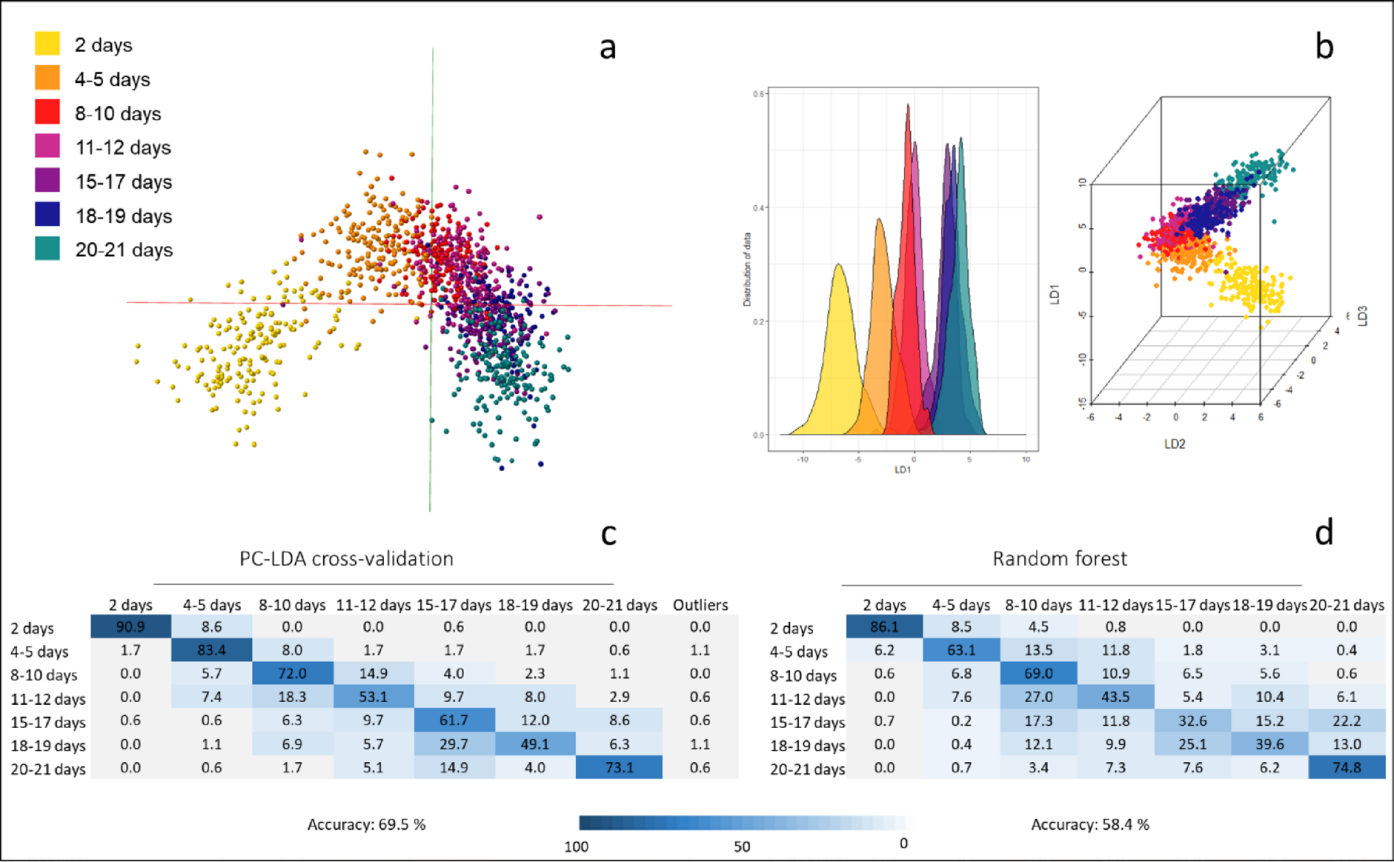
Discrimination of laboratory reared *Anopheles gambiae* mosquitoes by age. Anopheles gambiae mosquitoes were raised in the laboratory, fed sugar solution and/or blood meals before being killed by freezing and subsequent storage at room temperature. Specimens of seven age classes (2 days, 4–5 days, 8–10 days, 11–12 days, 15–17 days, 18–19 days and 20–21 days) were collected, with 175 females in each category. To explore the differences between the age groups the REIMS data was analysed using a combination of principal component and linear discriminant analysis (PC-LDA) and visualised through the Offline Model builder (OMB) software (**a**) and in the R environment (**b**) in form of 3D models and a kernel density plot. The PC-LDA separation was tested using cross-validation in OMB (using the option ‘Leave 20% out’ and a standard deviation of 5), resulting in an accuracy of 70% (**c**). Additionally, the data was analysed through random forest using a training/test data set split of 70 and 30%, leading to a total accuracy of 58% (**d**).

To establish whether blood meals and female physiological status would have a detrimental effect on the age-grading process, *Anopheles gambiae* mosquitoes were raised in the laboratory and offered sugar solutions as well as blood meals. The mosquitoes were snap frozen at different points in the gonotrophic cycle and then stored on silica gel at room temperature before being analysed with REIMS. A total of seven age classes (2 days, 4–5 days, 8–10 days, 11–12 days, 15–17 days, 18–19 days and 20–21 days) were collected, with 200 female specimens in each category. Samples from the sugar fed and blood fed cages were pooled within their age classes and 175 of each age category used to build the model with 25 retained as blinded samples. The raw data was imported into the Offline Model Builder (OMB) software and a model built using principal component-linear discriminant analysis (PC-LDA), which was subsequently tested through cross-validation (Fig. 1). The data matrix was exported and re-analysed using PC-LDA and random forest in the R environment. Despite adding blood-feeding as a potentially confounding variable, the age groups were arranged according to their increasing age, similar to the grouping seen in previous experiments^10^. Cross-validation of the PC-LDA model in OMB resulted in a total model accuracy of 70%; the blue shading displayed in the confusion matrix shows that samples were rarely misplaced in distant groups but rather mistaken for neighbouring age classes (Fig. 1C). Random forest analysis yielded a lower average accuracy of 58%, with the lowest accuracies lying in the 11–19 days age region (Fig. 1D). When classifications were assigned randomly to samples, separation of age classes failed and the resulting model reached a correct classification rate of only 18%, proving that age related differences in the mass spectral pattern are responsible for the separation when providing the correct class information (Supplemental Fig. 1). A subset of blood-fed and non-blood-fed mosquitoes from all age groups were kept separate as blind samples (*n* = 211) and were therefore not included in the model building data set. The model (Fig. 1) was exported to the OMB Recognition software and used to identify these samples; the percentage of correctly identified samples reached between 56% and 100% among different age classes (Supplemental Fig. 1).

**Fig. 2:**
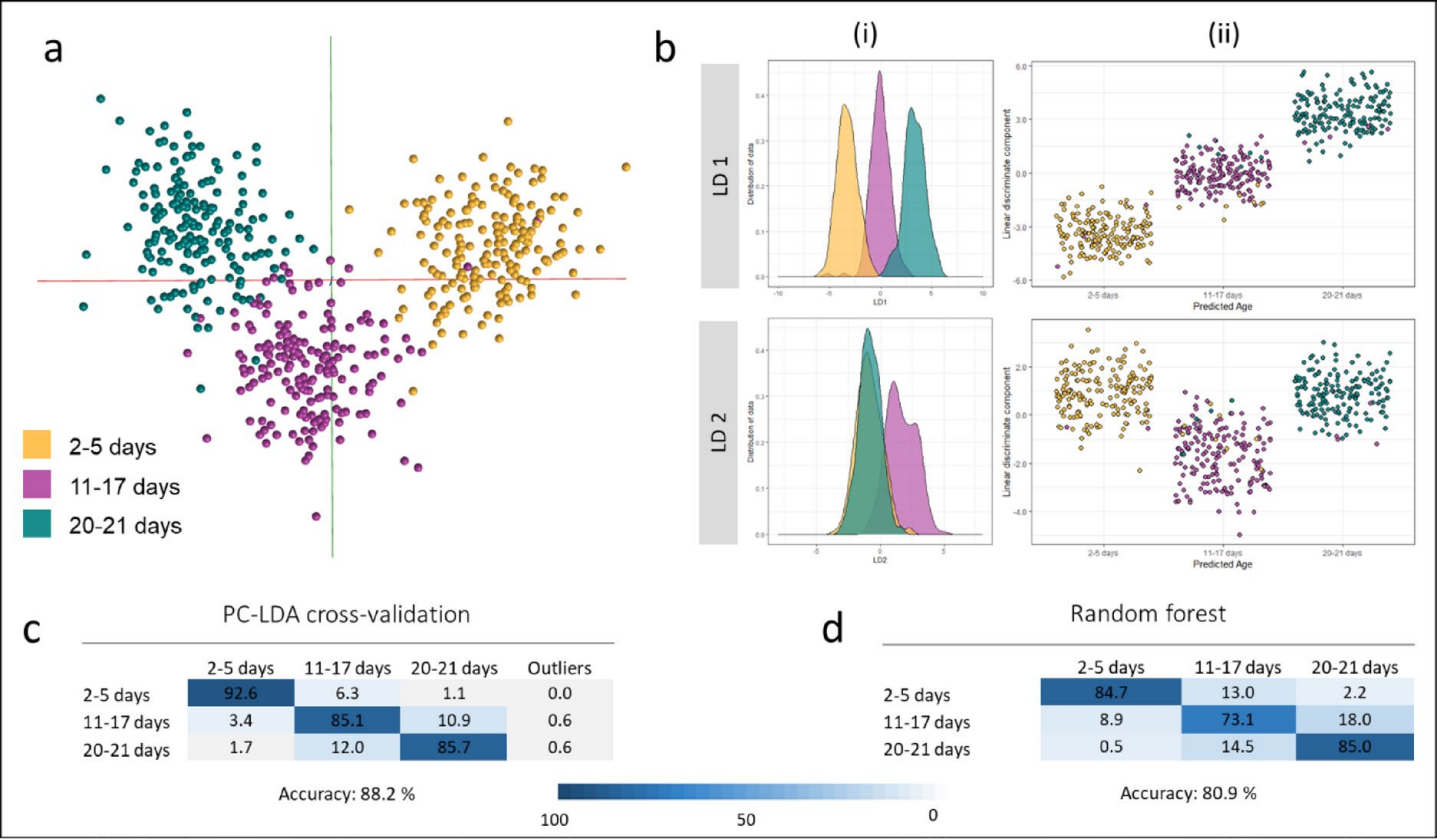
Discrimination of laboratory reared *Anopheles gambiae* mosquitoes into pooled age categories. To investigate the potential effect of class reduction on identification accuracy, age groups of the previous age model (Fig. 1) were either removed (8–10 days, 18–19 days) or combined. Samples from the first two age classes were randomly removed from the dataset to match the number of samples in the 20–21-day old class (*n* = 175). The new model with only three classes was again analysed using PC-LDA in OMB (**a**) and in R (**b**); the achieved separation is visualised as a 2D plot in OMB and as kernel density (i) as well as scatter plots (ii) based on linear discriminants 1 and 2 in R. Cross-validation of the 3-class model leads to an improved identification accuracy of 88% (**c**). Random forest analysis, again based on a 70/30% split of the data into training and testing data sets, also benefited from the class reduction and correctly identified 81% of the samples (**d**).

Given that age is a continuous variable, the overlap between adjacent age classes is unsurprising. For many field-related questions, separation into seven age brackets might not be required. Hence age groups from the previous model were either removed or combined to reduce the complexity of the identification model. The data was re-analysed through PC-LDA in OMB and R as well as random forest. Reduction of the number of age classes had a beneficial effect on the accuracy of the models, increasing the accuracy of the cross-validated model from 70 to 88% and the random forest accuracy from 58 to 81% (Fig. 2). As above the model was rebuilt with randomly assigned classifications and again the separation of age classes failed; as expected for three classes, the correct classification rate received through cross-validation only reached 33% (Supplemental Fig. 2). To determine whether blood-fed specimens negatively affected the separation, a five-class model was built without blood-fed mosquitoes and compared to the mixed sample set (Supplemental Fig. 3). Despite adding to the complexity of the data set, blood-feeding did not have a negative impact on the classification accuracy; the model without blood-fed specimens reached an accuracy of 82%, the model including the blood-fed mosquitoes reached 83%.

### Ovarian age can be reliably estimated using REIMS

**Fig. 3:**
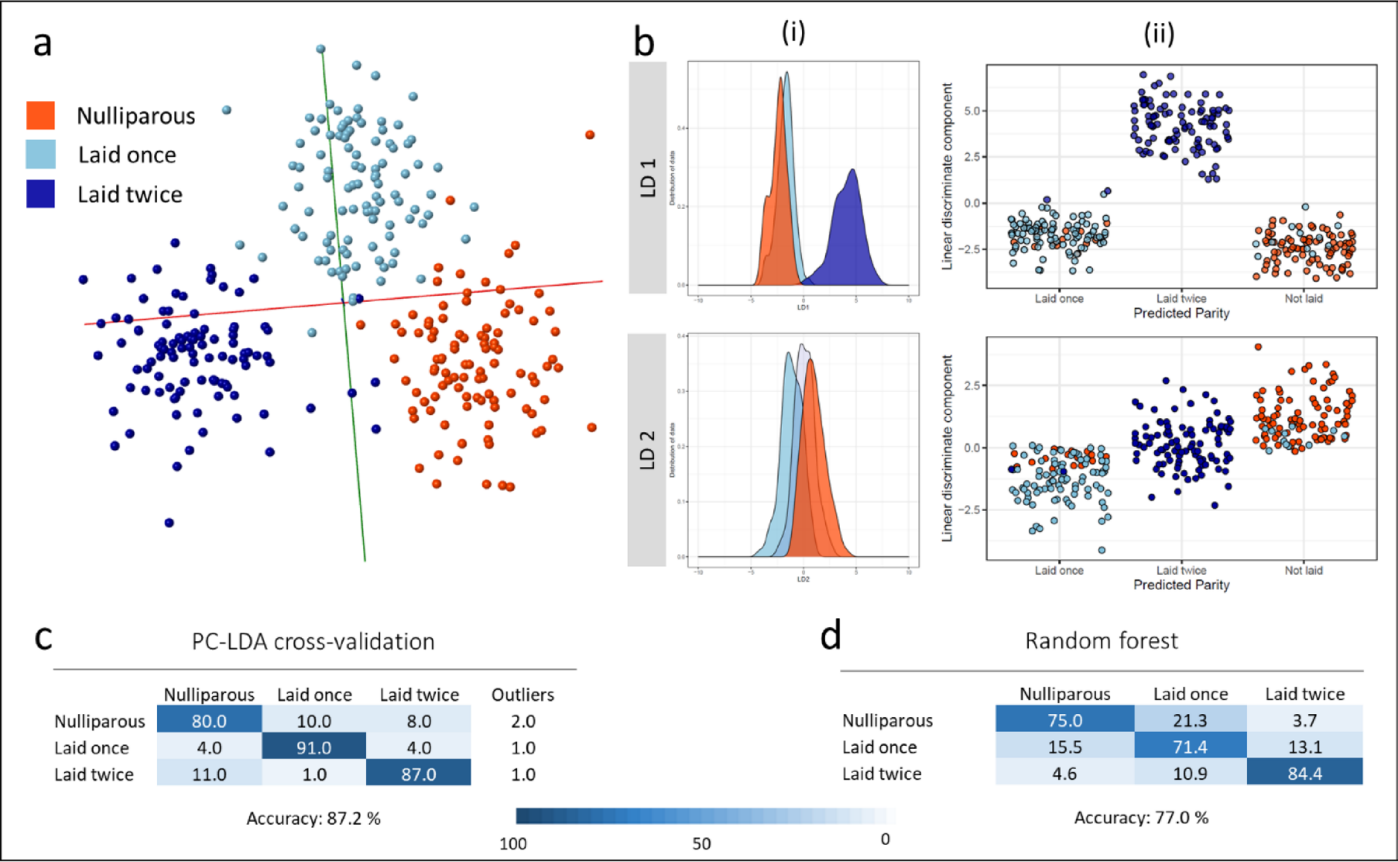
Discrimination of laboratory reared *Anopheles gambiae* mosquitoes by number of gonotrophic cycles. Mosquitoes that had been offered blood meals and kept separate to observe oviposition events were separated into three different categories: nulliparous (*n* = 100), laid eggs once (*n* = 100) and laid eggs twice (*n* = 100). PC-LDA based separation of the mosquitoes according to number of egg laying cycles is visualised as a 2D plot in OMB (**a**) and kernel density (i) and scatter plots (ii) in R (**b**). Both depict a clear separation of the group with two oviposition events along LD 1, while LD 2 aids the separation of the nulliparous from the group that had laid eggs once. The model built in OMB was cross-validated, achieving a classification accuracy of 87% (**c**). Using random forest, 77% of the samples in the test data set were correctly identified (**d**).

An alternative to establishing the chronological age is to assess the ovarian age, or number of egg laying cycles. To evaluate the ability of REIMS to distinguish mosquitoes that had completed different numbers of gonotrophic cycles, *An gambiae* females were offered blood meals and separated to oviposit individually; those that laid eggs were also offered a subsequent blood meal. In this way three separate groups of mosquitoes were obtained: nulliparous (i.e. had not laid eggs), mosquitoes that had laid eggs once and females that went through two oviposition cycles. This categorisation was used to group samples for REIMS analysis and model building (Fig. 3). The mosquitoes with two oviposition events were separated first along linear discriminant (LD) 1, whereas LD 2 further aided the separation of the nulliparous mosquitoes from the specimens that had laid eggs once (Fig. 3A,B). The cross-validated PC-LDA model in OMB was able to distinguish nulliparous mosquitoes and mosquitoes that had laid eggs once or twice with an overall accuracy of 87% (Fig. 3C). Random forest analysis reached an accuracy of 77% (Fig. 3D). The number of egg laying cycles, rather than number of blood meals taken is driving the model separation as the mosquitoes in the nulliparous group comprised a mixture of females that did not blood feed and those that blood fed but did not lay eggs; similarly the group that laid one egg batch contained mosquitoes that had received one or two blood-meals. Rebuilding the model with randomly assigned classifications lowered the correct classification rate to only 31% (Supplemental Fig. 4). The model built in OMB (100 PCs) was also tested using blind samples from all three parity groups; the percentages of correctly identified samples reached between 75 and 94% (Supplemental Fig. 4).

### REIMS accuracy is maintained in models built on field collected mosquitoes

**Fig. 4:**
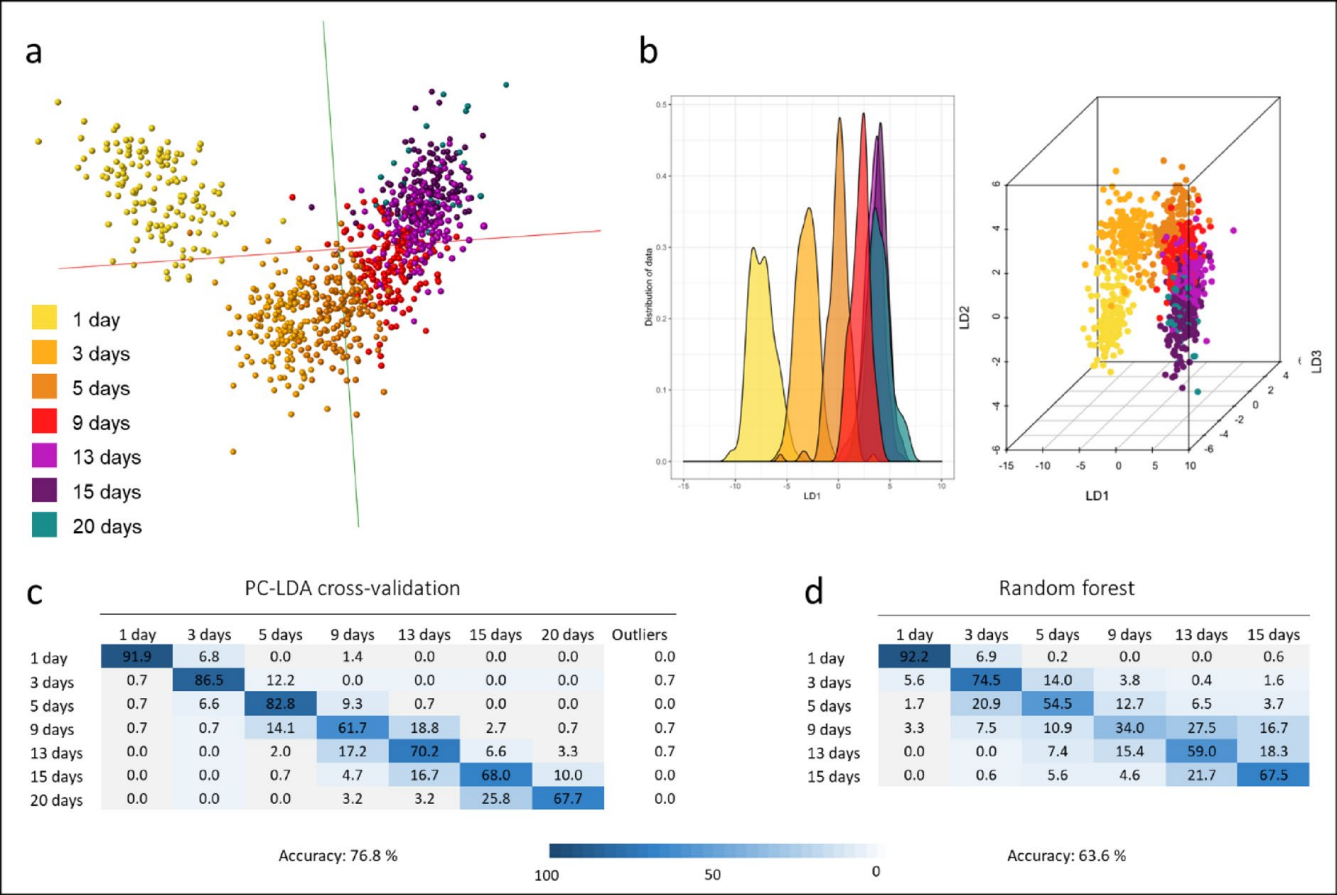
Discrimination of *Anopheles gambiae *mosquitoes collected from natural breeding sites as larvae and reared in the insectary by age. Mosquito larvae were collected from three different locations in Burkina Faso and reared in the insectary until they reached certain ages (1, 3, 5, 9, 13, 15 or 20 day old post emergence). After harvesting the adult specimens, they were again snap frozen and stored dry at ambient temperature until analysis. PC-LD analysis revealed a similar picture as seen in (Fig. 1), both in OMB (**a**) and R (**b**), separating continuously according to chronological age with a distinct separation of the young mosquitoes. Validation of the PC-LDA based model reached an identification accuracy of 77% (**c**), the random forest approach led to an average accuracy of 64% (**d**). Number of samples per age class: 1 day (*n* = 148), 3 day (*n* = 148), 5 day (*n* = 151), 9 day (*n* = 149), 13 day (*n* = 151), 15 day (*n* = 150), 20 day (*n* = 31). For random forest analysis the 20-day class was removed due to low sample numbers; equal sample numbers (*n* = 148) were used for the remaining six classes.

Mosquitoes used in the above experiments were from laboratory strains, maintained under strictly controlled environmental conditions. To determine if the accuracy of REIMS was maintained when analysing *An. gambiae* mosquitoes sampled from their natural habitat, mosquito larvae were collected from three different villages in the Cascades region of Burkina Faso and then reared in a local insectary until they reached a specific age. Mosquitoes were harvested at 1 day, 3 day, 5 day, 9 day, 13 day, 15 day or 20 day old post emergence; each age category consists of mosquitoes from all three locations to capture variation in larval breeding conditions. PC-LD analysis revealed a distribution similar to separations achieved previously with laboratory strains, leading from the youngest mosquitoes continuously to the oldest mosquitoes, with the 1-day old ones exhibiting the most distinct separation (Fig. 4). Repeated PC-LDA in R revealed the same separation within a 3D space displaying LD 1, LD 2 and LD 3. Cross-validation of the PC-LD model led to an accuracy of 77%, which exceeds the accuracy obtained in laboratory mosquitoes (70% Fig. 1C). The averaged accuracy of ten random forest runs reached 64%. As with previous models, misclassified samples were largely assigned to the adjacent age brackets. Separation of age classes failed entirely when classifications were assigned randomly, reaching a correct classification rate of only 22% (Supplemental Fig. 5). Samples (*n* = 264) that had not been included in the model building process were used as blind samples. The OMB model with the 77% accuracy was exported to the recognition software and 63% of the blind samples were correctly identified, 30% of identifications were in the neighbouring age brackets and 7% of samples were wrongly identified (Supplemental Fig. 5).

**Fig. 5:**
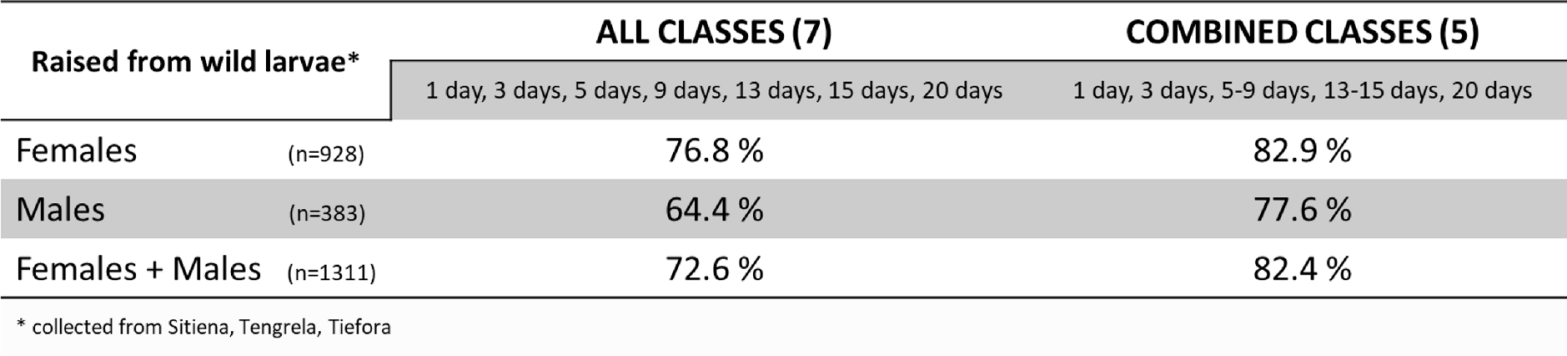
Comparison of identification accuracies of PC-LDA models including seven or five age classes based on either all male, all female or mixed sample sets. A variety of PC-LDA models were built in OMB based on sample sets differing in specimen sex and number of age classes. Number of samples in each age category specified for all three models. Females only: 1 day (*n* = 148), 3 day (*n* = 148), 5 day (*n* = 151), 9 day (*n* = 149), 13 day (*n* = 151), 15 day (*n* = 150), 20 day (*n* = 31). Males only: 1 day (*n* = 60), 3 day (*n* = 60), 5 day (*n* = 60), 9 day (*n* = 59), 13 day (*n* = 60), 15 day (*n* = 59) or 20 day (*n* = 25). Females and Males mixed: 1 day (*n* = 208), 3 day (*n* = 208), 5 day (*n* = 211), 9 day (*n* = 208), 13 day (*n* = 211), 15 day (*n* = 209) or 20 day (*n* = 56).

Male mosquitoes were also analysed through REIMS. A variety of models were built using this extended data set: models separating age groups that were only based on females, some that were only based on males as well as models that included both male and female mosquitoes. Additionally, age classes were reduced from seven to five classes by combining the 5- and 9-day old mosquitoes and the 13- and 15-day old mosquitoes. A comparison of the accuracies achieved for the models based on different sample sets and age classifications is presented in (Fig. 5). Note that the male only model was developed using a smaller number of samples than available for the female only model.

### Whole mosquitoes are not essential for approximation of mosquito age

There may be circumstances where it is desirable to retain part of a mosquito for follow up molecular work, or to dissect specific tissues for infection studies. We therefore determined whether a robust age grading model could be generated using only the abdomen. Despite using less biomass, the mosquito abdomens still produced sufficient aerosol and signal intensities to build an age separation model with five classes (1 day, 3 days, 5–9 days, 13–15 days, 20 days), which reached 67% accuracy (Supplemental Fig. 6). PC-LDA separation based on randomly assigned classes reached only 35% accuracy (Supplemental Fig. 7). The initial accuracy was slightly lower than for the full body samples, but this may be due to the use of a smaller sample set.

### Adults raised in semi-field conditions can be accurately aged

**Fig. 6:**
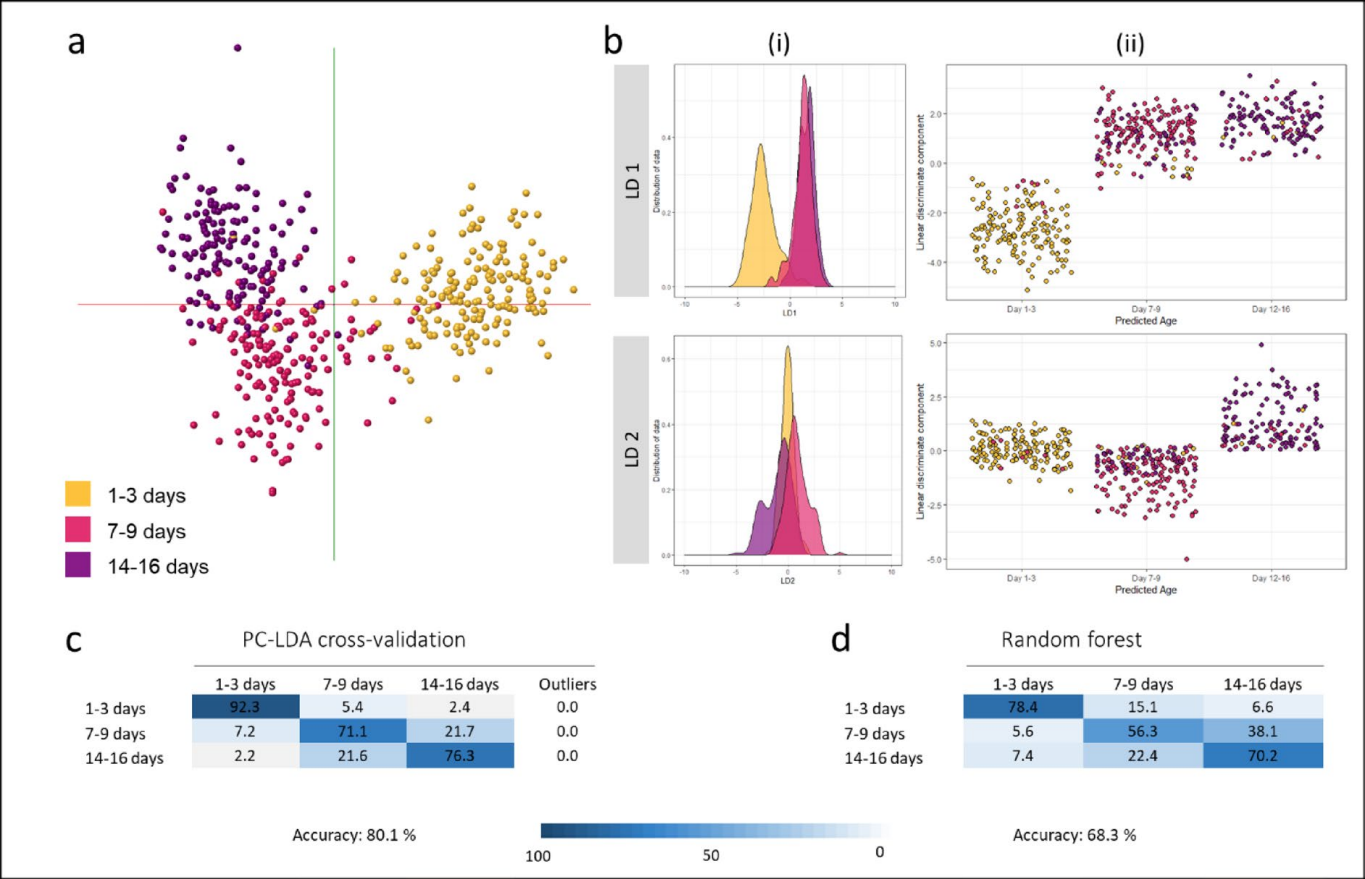
Discrimination of *Anopheles gambiae* mosquitoes collected as adults in semi-field stations (and big cages within) by age. To incorporate the effect of the natural environment mosquitoes were reared in semi-field stations and large cages within this semi-field set-up. After combining the female specimens from both rearing conditions and adjusting the age categories to three classes (1–3 days, *n* = 168; 7–9 days, *n* = 166; 14–16 days, *n* = 139), PC-LDA models were built in OMB (**a**) and within the R environment (**b**). Equal sample numbers (*n* = 139, for each class) were used for random forest analysis. The achieved separations are visualised as a 2D model in OMB (**a**) and kernel density (i) and scatter plots (ii) in R. Despite the increased complexity of the sample set, cross-validation of the PC-LDA model led to an identification accuracy of 80% (**c**), whereas random forest analysis accurately identified 68% of the samples (**d**).

While the larvae in the above experiments had been collected from three different villages and multiple breeding sites, the adults had still been raised in insectaries, and hence under conditions of reduced environmental variation than would be encountered in nature. To imitate the natural environment, another batch of mosquito larvae were allowed to emerge in large outside cages and semi-field stations (SFS) and supplied with sugar and blood sources for feeding. Females were collected at six time points from both the semi-field station and the large cages contained within the SFS. To increase sample numbers the mosquitoes from both set-ups were combined. The model built using all six age classes had only a correct classification rate of 56.8% due to poor separation of mosquitoes in age groups 9 day, 14 day and 16 day (Supplemental Fig. 8). Therefore, the age classes were combined and reduced to a total of three (1–3 day, 7–9 day, 14–16 day). PC-LD analysis revealed, in both OMB and R, that the youngest mosquitoes are separated first (LD 1), while LD 2 aids the separation of the two older groups (Fig. 6). The PC-LDA model built in OMB achieved an overall accuracy of 80%, while the random forest approach reached an accuracy of 68%. A model based on randomly assigned classes displayed a failed separation and a correct classification rate of only 33% (Supplemental Fig. 9) verifying the capacity of the model.

### Combining data from larval and adult collections improves the accuracy of the age grading models

**Fig. 7:**
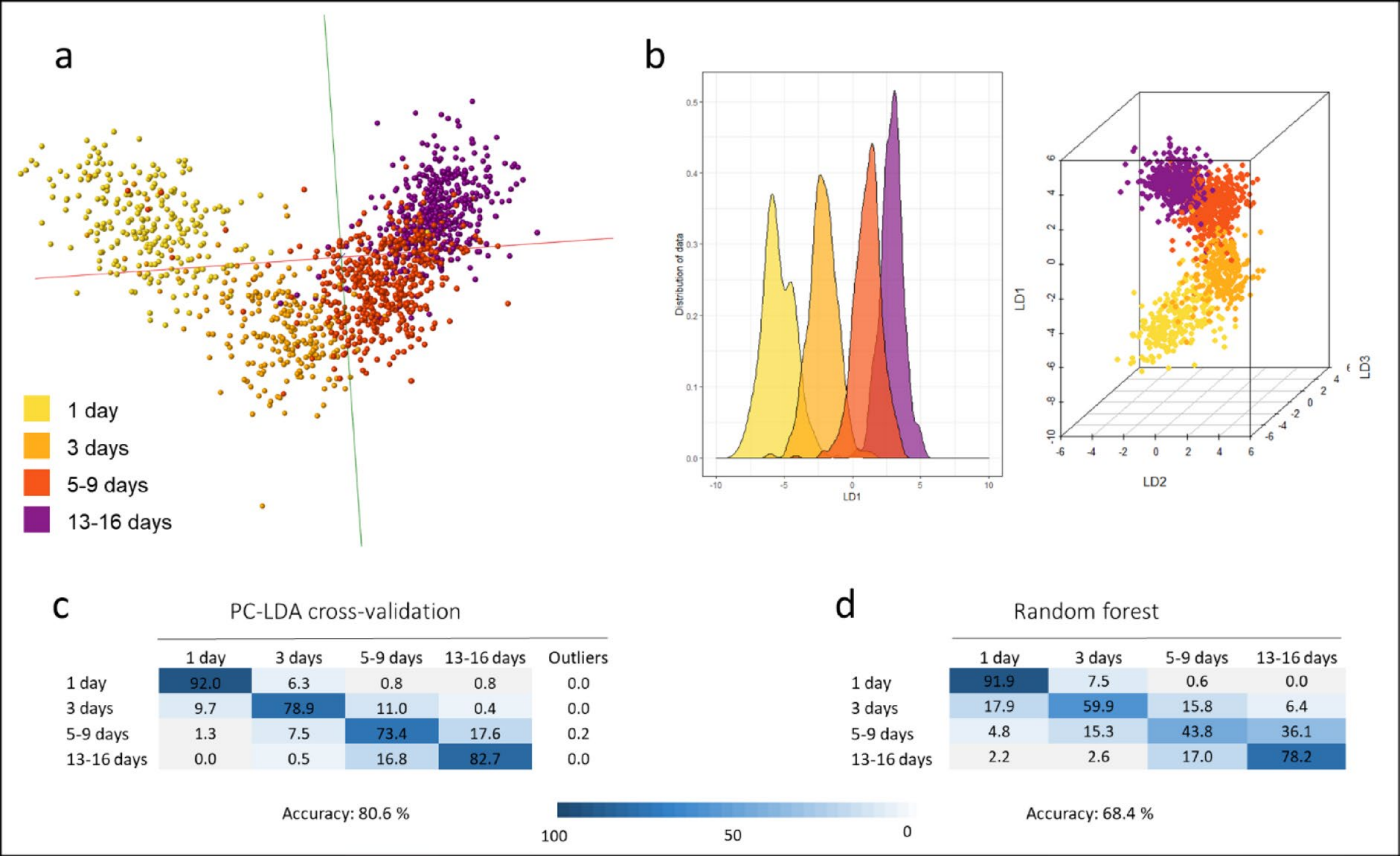
Discrimination of *Anopheles gambiae* mosquitoes into pooled age classes via a combined model from mosquitoes collected as larvae (in the Cascades region) and adults in semi-field stations (and big cages within) in the Hauts-Bassins region, by age. Specimens from two sample sets (Figs. 4, 6) were combined to increase complexity of the sample pool as well as sample size and were split into four age classes (1 day, *n* = 237; 3 days, *n* = 227; 5–9 days, *n* = 466 and 13–16 days, *n* = 440). Even sample numbers (*n* = 227) were used for random forest analysis. The age model was re-built with this combined sample set within OMB (**a**), using PC-LDA, and in R, using PC-LDA (**b**) and random forest. Cross-validation of the PC-LDA model yielded a correct identification rate of 81% (**c**); the average identification accuracy of the random forest analysis was 68% (**d**).

Classification models benefit from large sample numbers; we therefore combined the data collected from the mosquitoes raised in the semi-field station and large cages (Fig. 6) with the data from the mosquitoes that were raised in insectaries from wild larvae collections (Fig. 4). The combined data (1370 samples) was sorted into four age classes: 1 day, 3 day, 5–9 days and 13–16 days. The classification models were built in OMB and R, using PC-LDA as well as random forest; the latter was based on a data set with equal sample numbers across all age classes reaching a total of 908 samples (Fig. 7). This approach allowed for an increase in the samples numbers for classification whilst maximising the environmental variation in the data set (location, season, adult rearing conditions, species). Despite the larger amount of variation introduced by pooling across experiments, the models reached correct classification rates of 81% (PC-LDA, Fig. 7C) and 68% (random forest, Fig. 7D).

### REIMS can estimate age range of mosquitoes collected inside houses

A critical milestone to assess the utility of REIMS for age grading wild caught adult mosquitoes is to establish whether this methodology can reliably assign mosquitoes to epidemiologically relevant age groups when there is no prior knowledge about the mosquito life history (e.g. number of gonotrophic cycles, larval diet, insecticide exposure, pathogen infection). However, this experiment is challenging to do in the absence of a gold standard for aging mosquitoes. For most applications of age grading an estimate of population age, as opposed to the age of an individual mosquito, will suffice. Hence to determine whether, in principle, REIMS could detect differences in population age structure, adult female mosquitoes were collected from inside houses and then maintained in the insectary for zero, four or eight days. Using a classification model based on the 3 ‘age classes’ it was possible to show very distinct separation between the groups (Supplementary Fig. 10) indicating that the method could distinguish ‘older’ and ‘younger’ populations.

**Fig. 8:**
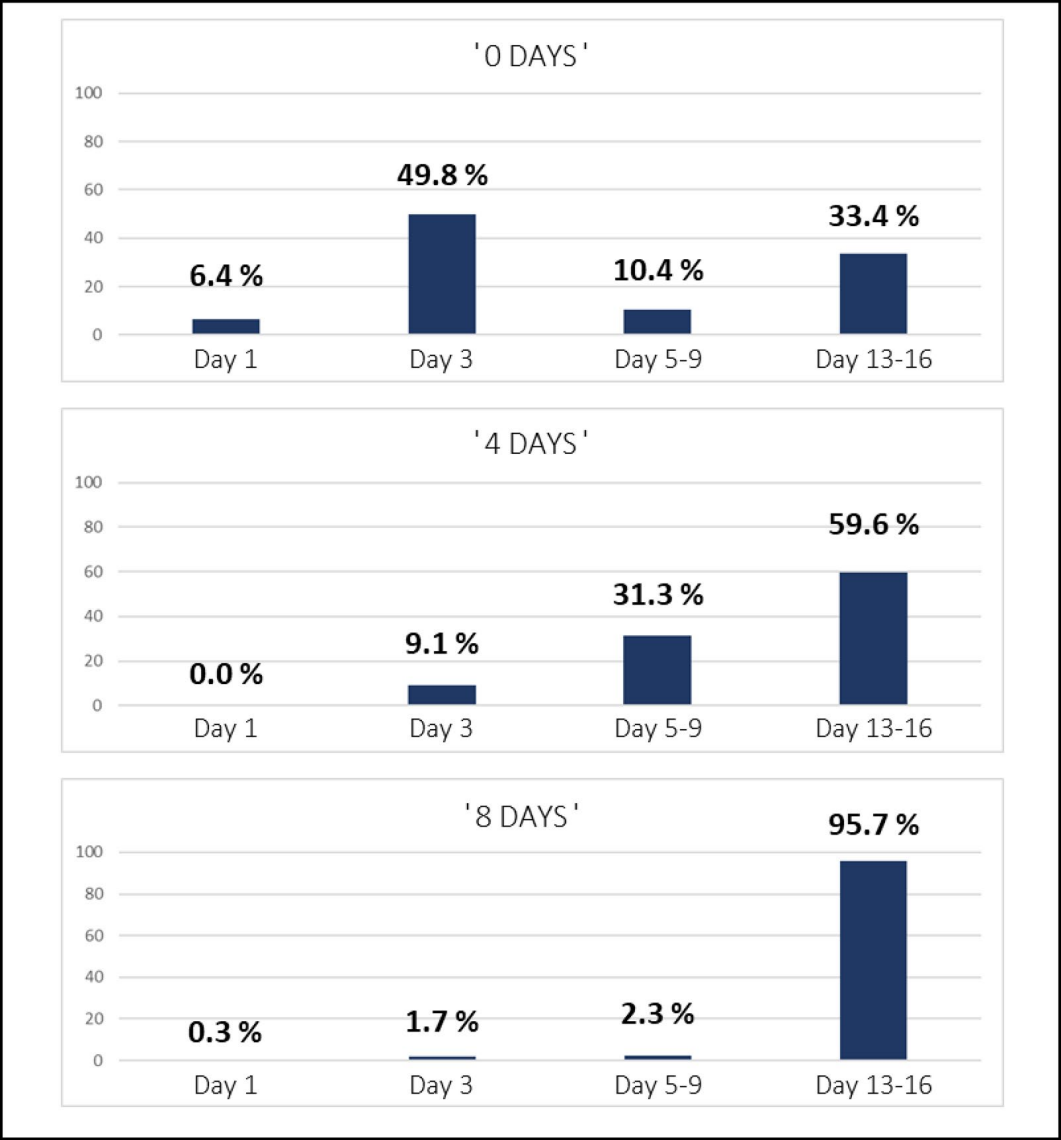
Age prediction of *Anopheles gambiae* mosquitoes collected as adults (and hence of unknown age) and retained in insectaries for 0, 4 or 8 days, using a model constructed from mosquitoes from larval and semi-field stations described in (Fig. 7). Adult female mosquitoes, collected from their natural environment, were used as unknown samples and identified using the combined age model (Fig. 7). The exact age was unknown; however, the mosquitoes were allowed to age further in the insectary to create three age groups: 0 (*n* = 299), 4 (*n* = 297) and 8 days old (*n* = 299). The previously built model was exported to the recognition software and used to sort the unknown samples into the four categories.

The experimental design could not distinguish how much of the variation between the three populations was determined by actual age differences in the sampled populations as opposed to the effect of prolonged stay of the wild mosquitoes in the insectary. To address this the wild caught adults were used as ‘unknowns’ in the combined data model built in OMB (Fig. 7) and their age estimated. As expected, the predicted age category increased with increasing days from collection to storage (Fig. 8). The largest percentage of ‘0 days’ samples were classified as 3 day (49.8%) followed by the oldest age category, 13–16 day (33.4%). After four days in the insectary (‘4 days’), the number of identifications in the 5–9 day and the 13-16-day categories increased, whereas none were identified as 1 day. In the ‘8 days’ sample group 96% of samples were identified as 13–16 day, with only 2% of samples classified as 1 or 3 days. This result shows that the REIMS model was capable of detecting changes in age distribution, even when using models constructed using samples from two different districts (maximum distance between collection sites is 111 Km) and via different collection methods.

### REIMS has potential for detecting additional entomological parameters of relevance to transmission potential

The ability to simultaneously detect multiple entomological indicators from individual mosquitoes would strengthen surveillance and enrich the evaluation of the impact of vector control products. The success of REIMS in distinguishing laboratory colonies of the *An. gambiae* complex has previously been demonstrated^10^. The larval collections in the current study were identified to species by PCR. Of a total of 570 samples from the three collection sites, 42.2% were *An coluzzii¸* 52.1% *An gambiae* and 5.6% *An arabiensis.* When a model was built using the full sample set, the accuracy of species identification was low (59.4%), possibly due to the unbalanced data set caused by a small sample size for *An. arabiensis*. Work is ongoing to rebuild the model and determine whether both species and age can be reliably ascertained using field collected samples.

We conducted a proof of principle experiment to determine the ability of REIMS to detect mosquitoes infected with *Plasmodium berghei* (a mouse malaria species commonly used in laboratory studies). A total of 449 mosquitoes, including specimens that had been fed a *P berghei* infected bloodmeal containing mature gametocytes twelve days prior, were used to train a model using PC-LDA (Supplemental Fig. 11). A subset of 50 mosquitoes from the same infection were retained for dissection and oocyst prevalence was confirmed at 98% with an average intensity of 66 oocysts/ mosquitoes (S.E.M. 6). Negative control non-infected mosquitoes were blood-fed on naïve, non-infected mice in parallel with experimental feeds. Cross-validation of the PC-LDA based model revealed an accuracy of 95.5%; using random forest classification, 96.4% of the samples were correctly identified. As expected, the classification accuracy was higher for the non-infected than the infected class; none of the samples in the ‘non-infected’ class are infected with *P. berghei*, the ‘infected’ class, however, might contain a small number of specimens without infection. Re-building the PC-LDA model with randomly assigned classifications showed that samples have a 50% chance to be assigned to one group or the other, indicating no clear group formation or separation with an accuracy rate of 44% (Supplementary Fig. 11).

## Discussion

We have shown that the accuracy of REIMS in age grading of laboratory reared mosquitoes reported earlier^10^ is largely retained when additional sources of intrinsic or extrinsic variation are introduced. As expected, and as observed with MIRS predictions^7^, accuracy of age grading increased when the number of age categories was reduced, with predictions of intermediate aging being less robust than very young or very old age categories. Encouragingly, a model built from samples derived from larval and adult collections from multiple sites in southwest Burkina Faso, and including a mixture of three species within the *An. gambiae* complex, was still able to classify mosquitoes into four age categories with an accuracy > 80%, thereby illustrating that models do not need to be built *de novo* for each mosquito collection. Further work is needed to determine how generalisable the models are when encountering greater sources of ecological, environmental, geographical or temporal variation than incorporated into the current study.

The gonotrophic cycle in Anopheles is an indicator of the time interval between bloodmeals or oviposition events and, whilst typically ranging from 2 to 4 days in *An gambiae* under optimal conditions^11^, can vary depending on season, temperature, species or other factors^12^. Given the concordance between ovarian age and number of bloodmeals, and the central role of blood feeding in disease transmission, ovarian age may be a more useful indicator of vectorial capacity than measurements of chronological age. Ovarian age can be measured by counting dilatations in ovarioles^4^ but this requires highly skilled personnel and is low in sample throughput meaning that in reality dissection based methods typically use a binary classification of parous or nulliparous^3^. Hence the finding that REIMS has the ability to accurately distinguish between nulliparous mosquitoes, mosquitoes that have laid once (blood fed once and thus potentially infected) and laid twice (blood fed twice and hence potentially transmitting pathogens) is significant. Further studies are needed to determine the accuracy of prediction of ovarian age under field conditions.

Studies on the age structure of populations typically focus on female mosquitoes given that males do not play a direct role in disease transmission. Nevertheless, the finding that models built with males only, or models built with mixed sexes have similar accuracy levels to the models built using females alone is encouraging. There is an absence of data on the longevity of male mosquitoes and yet this information would inform models evaluating certain intervention strategies such as targeting swarms^13^. The impacts on males and females may also differ for other vector control tools that target mosquito behaviour. For example, ITNs and Indoor Residual Spraying primarily target females, whereas Attractive Targeted Sugar Baits and larval source management target both sexes. If age structure analysis is to be used to assess intervention impacts, the ability to assess the impact on the population age structure of both males and females might prove important.

Ultimately, any age grading methodology would ideally be able to estimate the population age of mosquitoes using models trained on independent data sets. In a preliminary attempt to evaluate the ability of REIMS to meet this goal, we used the model developed using mixed larval and adult collections to estimate the age of mosquitoes collected from inside houses. In the absence of a gold standard for age grading it is not possible to directly assess the accuracy of the predictions, but we believe the initial results are promising. Mosquitoes sampled directly after collection from houses were distributed across all four age groups with the majority being found within the 3 day or > 12-day category (noting that predictions of intermediate age groups are typically more challenging as discussed above). As expected, as the length of time the mosquitoes were retained post collection increased, the average predicted age also increased. This result is a promising indication that REMIS may be able to detect a shift toward younger age distributions, following the implementation of vector control, for example.

A draft Target Product Profile recently developed for evaluation of infrared machine leaning based systems for entomological surveillance of malaria vectors^9^ provides a useful framework to benchmark the REIMS approach in relation to scope, performance and operational aspects. In terms of scope, we have now shown that, in principle, REIMS can identify mosquito age, species, and detect malaria pathogens although we have yet to fully evaluate simultaneous detection of these traits under field conditions. We have also not yet attempted to identify bloodmeal source using REIMS. At present, the instrumentation being used is expensive and probably equipped with an unnecessarily high specification; any further development of this methodology should prioritise evaluating lower resolution instruments to reduce the capital expense of adopting this method. However, once established, the price per sample, and rates of throughput are favourable compared to other age grading methods^10^.

The technical performance of REIMS appears to be broadly comparable with the DL-MIRS methodology^14^ with reliable detection of Plasmodium (with the important caveat that so far we have only tested on laboratory infections with much higher oocysts counts than typically found for *P. falciparum* in nature) and > 80% accuracy in classifying mosquitoes into three or more age groups. As REIMS is a destructive methodology, we evaluated whether partial bodies (in this case abdomens) could be used for age grading enabling additional molecular or parasite detection assays to be performed on the same individual. Initial results are promising; although the accuracy for the five class model was lower than obtained with whole mosquitoes (67% vs. 83%) possible explanations include the smaller sample size used for analysis of dissected mosquitoes versus whole mosquitoes (*n* = 630 vs. 1225), the reduced mass of material and/or variations in the complexity of the sample that affect the information richness of the ionised sample.

Favourable operational aspects of the REIMS approach include the speed of processing, minimal reagent requirements, plus the ability to use dried specimens (models in this study were built using mosquitoes stored on silica gel at room temperature from between 7 and 180 days) and we have previously shown that REIMs performs equally well on frozen and dried material (see 10).

Vectors must survive the extrinsic incubation period (EIP -the development time of pathogens within the vector) to be able to transmit parasites onward meaning that bites from younger vectors are not a risk to communities. One of the longer-term goals of developing a robust methodology to measure mosquito population age structures is to use this as a proxy for the efficacy of vector control interventions. For example, insecticide-based interventions are expected to reduce the life expectancy of adult mosquitoes and detection of shifts in age distributions toward younger profiles could be a useful assessment for efficacy. However, there remain important questions on how age structures shift throughout a season, how sensitive age structures are to weather events (that are likely different year- on- year), and how we can distinguish between the shift to a younger age distribution that is due to, for example, a preceding week of heavy rain, or an early phase of the transmission season, in contrast to the response (a true shift toward younger vectors) following the implementation of vector control. Comparing trial arms using age structure analysis would require an appreciation of these sensitivities. As we learn more about how climate-sensitive metrics such as EIP alter transmission probabilities, we can tailor approaches like REIMS to distinguish critical age groups in a spatially specific fashion. For example, in climes with longer EIPs, identifying the proportion of female mosquitoes that are over 10 days of age, and how this proportion is shifted by vector control, may be most critical. Whilst in shorter EIP climes, mosquitoes of 7 days of age and older may be important to target.

In conclusion the REIMS approach has the potential to be a useful addition to mosquito surveillance or in the assessment of the entomological impact of vector control. The next step in translating this to a field deployable intervention is to investigate options for lower cost back end hardware. In the meantime, a direct comparison between DL-MIRS and REIMS would be helpful for benchmarking purposes and to potentially identify use case scenarios where each methodology shows most promise.

## Methods

### Study sites

Experimental studies were conducted in the UK and Burkina Faso. Experiments in the UK were conducted in the LITE facilities of the Liverpool School of Tropical Medicine (LSTM) in temperature and humidity controlled insectaries (26 ± 2 °C and a relative humidity of 80 ± 10% under a L12:D12 h light: dark cycle with a 1-h dawn and dusk). In Burkina Faso, larvae were collected in August and September 2021 and September and October 2022 from four villages Sitiena (-4.80490°W, 10. 60366 °N), Tiefora (-4.54916 °W, 10. 62368 °N) and Tounmousséni (-4.98095°W, 10. 20376 °N ) (2022 only) in the Cascades region in the southwest of the country. Larvae were collected as 3rd or 4th instar and reared to adults at the Centre National de Recherche et de Formation sur le Paludisme (CNRFP) insectaries in Banfora. Semi Field Studies were performed at the Institut de Recherche en Sciences de la Santé (IRSS) in the north of Bobo-Dioulasso in Vallée du Kou village VK5 (4.4201°W, 11.3824°N).

### Mosquito collection and rearing

#### Laboratory colonies

The Kisumu strain of *An gambiae* s.s. was used for experiments at LSTM. Approximately 600 pupae were added to six cages and allowed to emerge. Any pupae that had not emerged 24 h later were discarded. A subsample of 100 mosquitoes were collected 2 days later (age group 2 days); four of the cages were then offered a blood meal (day 3) using human blood, procured from the non-clinical blood product stock of a UK blood bank, and a haemotek feeder with the remaining two cages being maintained on sugar only. The blood fed cages were re-fed a further two times (day 10 and day 17); on each occasion, females that had not blood fed were removed from the cage and discarded. Oviposition pots were provided on days 5 and 12. Fifty females were sampled from the blood fed and sugar fed cages at 2 days, 4–5 days, 8–10 days, 11–12 days, 15–17 days, 18–19 days and 20–21 days post emergence. All samples were snap frozen before being transferred to individual Eppendorf tubes with silica gel and stored for at least 7 days at room temperature before processing. The experiment was repeated a second time, using the subsequent generation of Kisumu. Mosquitoes from both replicates were pooled for analysis; mosquitoes from two consecutive days were combined to form 6 age groups. The experiment was designed to ensure that the sampled females contained a mixture of sugar and blood fed and gravid and non-gravid females at different ages to determine whether physiological status reduced the accuracy of the REIMS age grading.

In a separate experiment to determine whether REIMS could be used to assess the number of gonotrophic cycles female mosquitoes had undertaken (ovarian age, a potential proxy for biological age) mosquitoes were transferred to individual oviposition tubes after blood feeding. Briefly, approximately 2000 Kisumu pupae were transferred into a cage and the emerging adults were split into two groups, a blood fed and non-blood fed group. Mosquitoes were offered a blood meal on day 4 post emergence. Non-blood fed females were removed the following day and on day 6 females were aspirated into individual oviposition tubes (50 ml falcon tubes containing a dampened filter paper in the base of the tube to provide an egg laying substrate) for 72 h. A subset of 50 mosquitoes that had laid eggs and 50 that did not lay eggs were snap frozen and retained on silica gel. The remaining females were transferred back to a cage and blood feed again on day 10. The oviposition process was repeated as above with 50 mosquitoes that had completed two gonotrophic cycles stored on silica gel. The full experiment was repeated a second time, using a subsequent generation of Kisumu. In total, of 417 females offered a blood meal across both experiments, 76% blood fed and 230 of these (73%) oviposited, 113 (49%) in round one only and 117 (51%) in round one and two.

#### Larval collections

Anopheles larvae were collected from multiple aquatic habitats in each of the three villages and transferred to the Banfora insectaries. Larvae were maintained in chlorine-free borehole water and fed with cat food (Friskies ^®^). Pupae were transferred to cages (separate cages for collections from each of the three villages) and males and females were kept together under insectary conditions (27 ± 2 °C temperature, 70–80% humidity with 12:12 h light and dark cycle) with glucose (5%) provided *ad libitum*. Approximately 50 females and 20 males were aspirated from the cages on days 1, 3, 5, 9, 13 and 15 post emergences. Legs were removed for species ID and the remaining mosquitoes stored individually in Eppendorf tubes with silica gel. The remaining mosquitoes were harvested at 20 days.

In a separate experiment, the larval collections were repeated the following year, transferred to the Banfora insectaries and raised to adults as above but in this experiment, the abdomen was separated from the head and thorax prior to storage. Abdomens were used for REIMS analysis and the remaining parts for the species ID.

#### Species identification

Mosquito legs were used for mosquitoes’ identification. DNA extraction consisted of placing the six legs in a 1.5 mL microcentrifuge tube with 30 µL of STE (100 mM NaCl; 10 mM Tris-Cl, pH 8.0; 1 mM EDTA). The samples were heated at 95 °C for 15 min to release DNA, then cooled to room temperature and centrifuged to pellet debris. The supernatant containing DNA was transferred to a fresh tube and stored at -20 °C for further PCR-based species identification following the method of^15^.

#### Plasmodium berghei infection and analysis

*P. berghei* ANKA 2.34 parasites were maintained in 6- to 8-week-old female CD1 mice (Harlan) by mechanical passage (up to a maximum of eight). If required, hyper-reticulosis was induced 3 days before infection by treating mice intraperitoneally (i.p.) with 200 µL phenylhydrazinium chloride (PH; 6 mg/mL in PBS; ProLabo UK). Mice were infected i.p. and infections were monitored using Giemsa-stained tail blood smears as described previously^16^.

To infect mosquitoes, two groups of five mice were housed in individually ventilated cages. One (blood-feed only control) cage was left uninfected, whereas the remaining five mice were PH-treated, and 3 days later infected i.p. with 10^6^ *P. berghei* ANKA 2.34. Three days post-infection, animals were anesthetized, and > 500 female *Anopheles stephensi* mosquitoes allowed to blood feed on each mouse. Twenty-four hours later, unfed mosquitoes were removed. Mosquitoes were maintained on 8% (w/v) fructose, 0.05% (w/v) p-aminobenzoic acid at 19–22 °C, and 50–80% relative humidity. Day 12 post-feeding, 50 individual mosquito midguts were dissected and oocyst intensity and prevalence observed by standard phase microscopy and recorded to establish actual infection levels. The remaining ∼450 mosquitoes were examined by REIMS as described below.

#### Semi field experiments

*Anopheles* larvae were collected from different breeding sites located in Vallee de Kou VK7 village, in Bama between September and October 2021 and transferred to the field insectaries at IRSS and reared until pupae stage. Pupae were released into a newly refurbished semi-field station (SFS) located in the same village. The SFS is a fully screened house divided in several compartments separated by a corridor as described^17^. The climatic conditions including temperature, light, humidity, wind speed within the SFS were the same as outdoor conditions. The whole SFS is protected by a surrounding narrow water-filled channel to exclude ants and other predators. The release was done in one compartment refurbished for the experiment. In this compartment four claypots, containing cotton wool soaked in sugar water (5% glucose) were provided to serve as refuge of mosquitoes. Potential oviposition sites were emptied every 3 days to discard any larvae; in this way the age of the adults collected in the SFS could be ascertained with an accuracy of +/- 1 day from the date of addition of pupae. Approximately 50 adult females were collected from the SFS by aspiration on day 1, 3, 7, 9, 14 and 16 post 1st emergence. Mosquitoes were stored in individual Eppendorfs containing silica gel.

In order to mitigate against low recapture rates in the SFS, a separate subset of 150 pupae were released into a big cage each round ((2 × 2 × 2 m). Pupae were introduced and aspirated on the same day into both the big cage and SFS. Mosquitoes in the big cage did not have access to a blood meal.

#### Adult collections

Approximately 900 adult female mosquitoes were aspirated from inside homes in Tengrela using electric aspirators. Collections were performed between the hours of 6h00 AM and 9h00 AM in September and October 2021. Aspirated mosquitoes were stored in paper cups and then transported to the Banfora insectaries conserved in a coolbox. At the insectary mosquitoes from the same location were pooled and transferred into cages. Three hundred mosquitoes were snap frozen and then transferred to individual Eppendorfs with silica gel on the day of collection. The remaining mosquitoes were retained in cages with a source of sugar before being harvested at either day 4 or day 8 post collection.

#### REIMS analysis

Samples were analysed via a rapid evaporative source (REIMS, Waters, Wilmslow, UK) attached to a Synapt G2Si instrument ion mobility equipped quadrupole time of flight mass spectrometer (Waters, UK). The specimens were burned/evaporated using a monopolar electrosurgical pencil (Erbe Medical UK Ltd, Leeds, UK), which was connected to a VIO 50 C electrosurgical generator (Erbe Medical UK Ltd, Leeds, UK), providing electrical current, and to the source inlet via plastic tubing. A black conductive rubber mat, placed underneath the samples, acted as a counter electrode and facilitated the flow of electric current. To avoid inhalation of fumes during analysis, the burning process was performed within a fume box (Air Science, Lydiate, Merseyside, UK). Insects were analysed using a 40 W setting on the generator and the cutting option of the pencil. To increase conductivity and to protect the counter electrode during analysis, specimens were placed on a piece of glass microfibre paper (GFP, GE Healthcare Whatman) on top of a wet paper surface (moistened with MilliQ water).

While burning the entire biomass of single specimens, the aerosol was aspirated through the pencil and the attached 3 m long tubing into the REIMS source, using a nitrogen powered venturi valve on the source inlet. To increase the aerosol capture, a wide bore piece of plastic tubing was additionally placed over the tip of the electrosurgical pencil. A whistle incorporated into the Venturi tube guided the aerosol as well as a lock mass solution of leucine enkephalin (Waters, UK) in propan-2-ol (CHROMASOLV, Honeywell Riedel-de-Haën) into the source. This also filters the incoming aerosol to prevent larger particles from entering the inlet capillary. Inside the source, the ionised particles were declustered through contact with a heated impactor (Kanthal metal coil at 900 °C).

Mass spectra were acquired in negative ion mode at a rate of 1 scan per second between 50 m/z and 1200 m/z. The sample cone and heater bias were set to 60 V. Instrument calibration was performed daily in resolution mode using a 0.5 mM solution of sodium formate (flow rate 50 µl/min). The lock mass solution (0.4 µg/ml, leucine enkephalin in IPA) was continuously introduced during sample analysis at 30 µl/min. For each experiment, samples were analyzed in a formally randomised order on one or more days.

#### Data analysis

The raw mass spectra were imported into the model building software package Offline Model Builder (OMB-1.1.28; Waters Research Centre, Hungary), which allows separation of sample groups (classifications) based on principal component analysis (PCA) and linear discriminant analysis (LDA). Data were additionally analyzed using R (version 4.2.1)^18^ and the R Studio environment^19^, by PCA and LDA, as well as random forest analysis.

For Offline Model Builder, the burn events of the analyzed specimens were defined individually, summing all the MS scans within each chosen area. The option to create one spectrum per sample was selected. Other pre-processing parameters included the intensity threshold, which was set between 4e5 and 9e5 (depending on the background baseline), spectra correction using the lock mass (leucine enkephalin, 554.26 m/z) and background subtraction. To reduce the complexity of the mass spectral data, all acquired data points from 50 to 1200 m/z were combined into mass bins, each 0.1 m/z wide. Subsequent model calculation was based on principal component and linear discriminant analysis (PCA-LDA).

The models built by Offline Model Builder were cross-validated (leaving out 20% of data, for outliers the standard deviation multiplier was set to 5) to obtain the correct classification rate, as well as the number of failures and outliers and a matrix displaying the number of correctly and incorrectly identified samples of each classification. To additionally test discrimination, sample classifications were randomised and the data were re-analyzed, the expectation being a random distribution of samples and the inability to achieve separation.

For further analysis with R, the data matrix of each model was exported as a .csv file from Offline Model Builder, containing information about classification and the relative intensities for every mass bin. The matrices were used to perform random forest analysis in R using the package ‘randomForest’^20^. The data sets were randomly split into a training set (approx. 70% of the data) and a test set (approx. 30% of the data). Random forest results are displayed in form of confusion matrices. Trees were conducted 10 times for every model (using a different, randomly selected subset of samples for training and testing every time); the numbers of correctly identified and confused samples were turned into percentages and averaged. The optimal number of trees and mtry value were determined during the first analysis of each model and kept the same for each repeated analysis. A second R package, called ‘randomForestExplainer’^21^, was used to identify the most informative bins/ions that were driving class separation. PCA-LDA was also performed within the R environment, using the in-built package ‘stats’ and the package ‘MASS’^22^ and results visualized in form of kernel density plots and 2D- and 3D-scatter plots created using ‘ggplot2’^23^ and ‘scatterplot3d’^24^. PC-LDA based models were built with a quarter of the maximum number of principal components (PCs) unless stated otherwise. In OMB, models were built with a maximum of 100 PCs.

## Electronic supplementary material

Below is the link to the electronic supplementary material.

Supplementary Material 1

## Data Availability

The analysis code, and datasets generated during and/or analysed during the current study are available here https://doi.org/10.17638/datacat.liverpool.ac.uk/3012.
