# Supplementary material for: Rapid evaporative ionisation mass spectrometry can age field caught Anopheles gambiae malaria vectors
Source: Sci Rep. 2025 Jun 2;15:19342. doi: 10.1038/s41598-025-03779-x (PMC12130540; doi:10.1038/s41598-025-03779-x)
Supplement: Supplementary file 1 — Supplementary Material 1 [file 41598_2025_3779_MOESM1_ESM.docx]

SUPPLEMENTARY MATERIALS

**Supplemental Figure 1:** ***Randomised model of laboratory reared* Anopheles gambiae *mosquitoes and identification of blind sample set.***


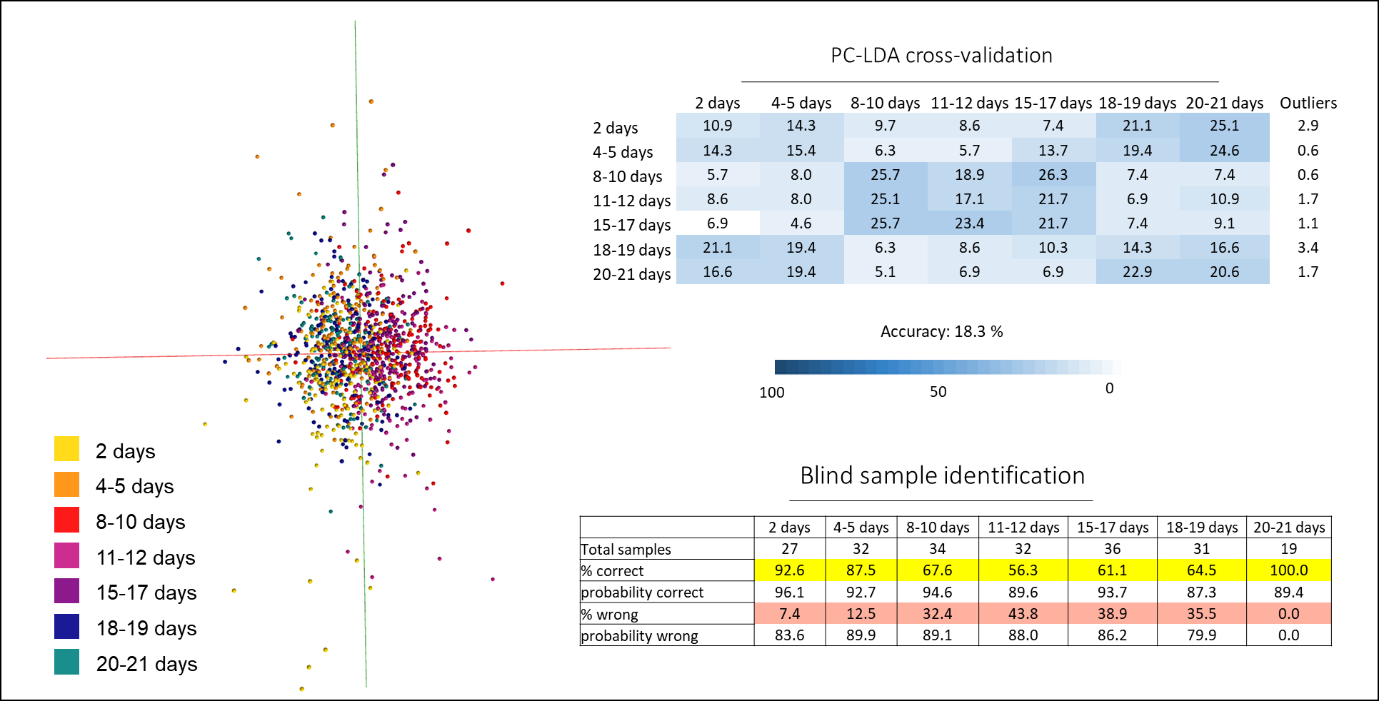
*Classifications were randomly assigned to the samples and the model with seven age classes re-built and cross-validated. The model achieved an accuracy of only 18 %; the age classes could not be separated. Using the model based on the correct mosquito age, blind samples were identified using a recognition software; between 56 and 100 % of samples per class were correctly identified.*

**Supplemental Figure 2:** ***Randomised model of laboratory reared* Anopheles gambiae *using three age classes.***

***
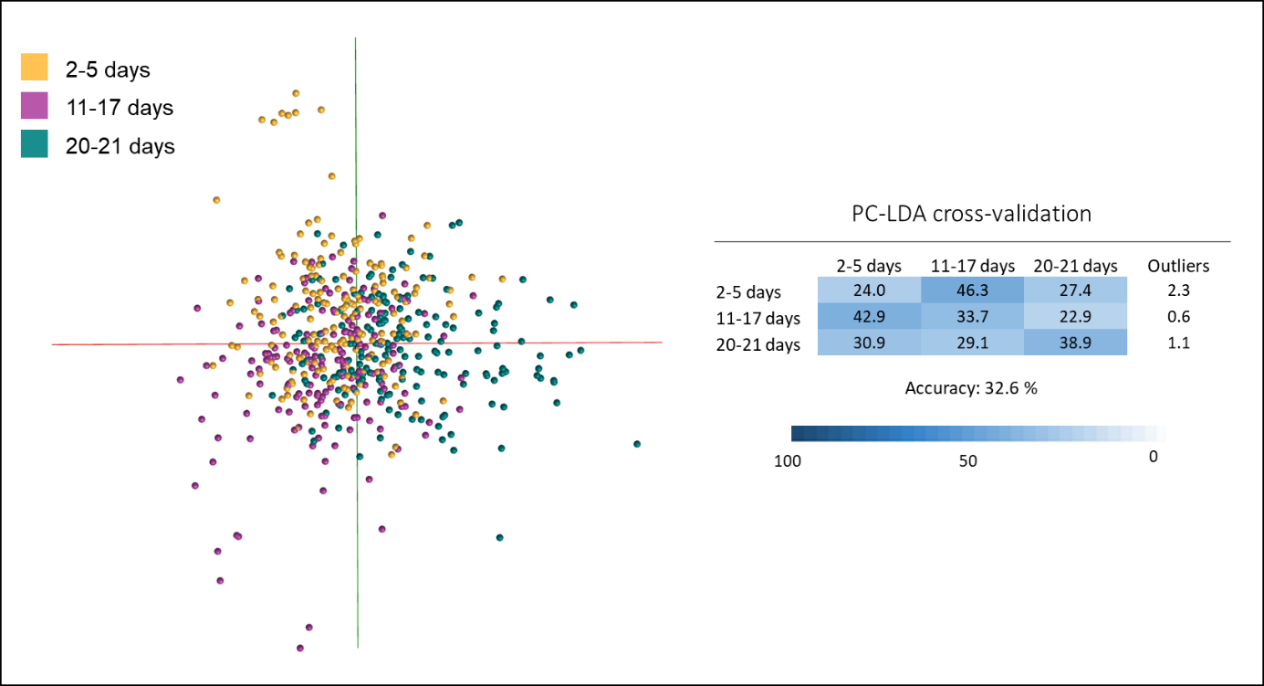
****The three age classifications were randomly assigned to the samples and the model was re-built and cross-validated. The tested model achieved an accuracy of only 33 %.*

**Supplemental Figure 3:** ***Comparison of the laboratory reared* Anopheles gambiae *models (5 age classes) with (top matrix) and without blood-fed mosquitoes (bottom matrix).***


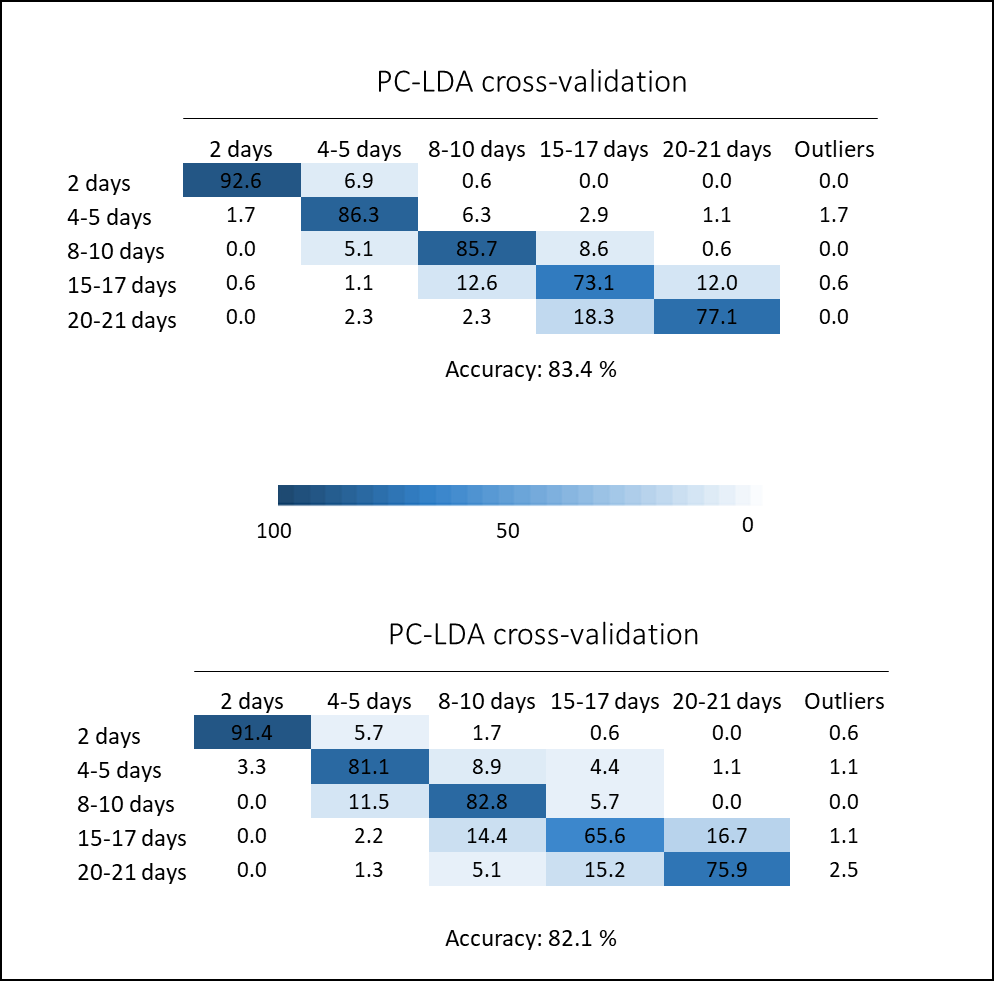
*To examine whether blood-feeding negatively affects the accuracy of age models, two models were built based on samples from the same experiment: one including blood-fed and sugar fed specimens (top panel,n=1225) and one with sugar fed only (bottom panel, n=612). Including blood-fed mosquitoes did not negatively affect model building or decrease age-grading accuracy.*

**Supplemental Figure 4:** ***Randomised model of the parity model (75 PCs) and blind sample identification using the recognition software (100 PC model).***


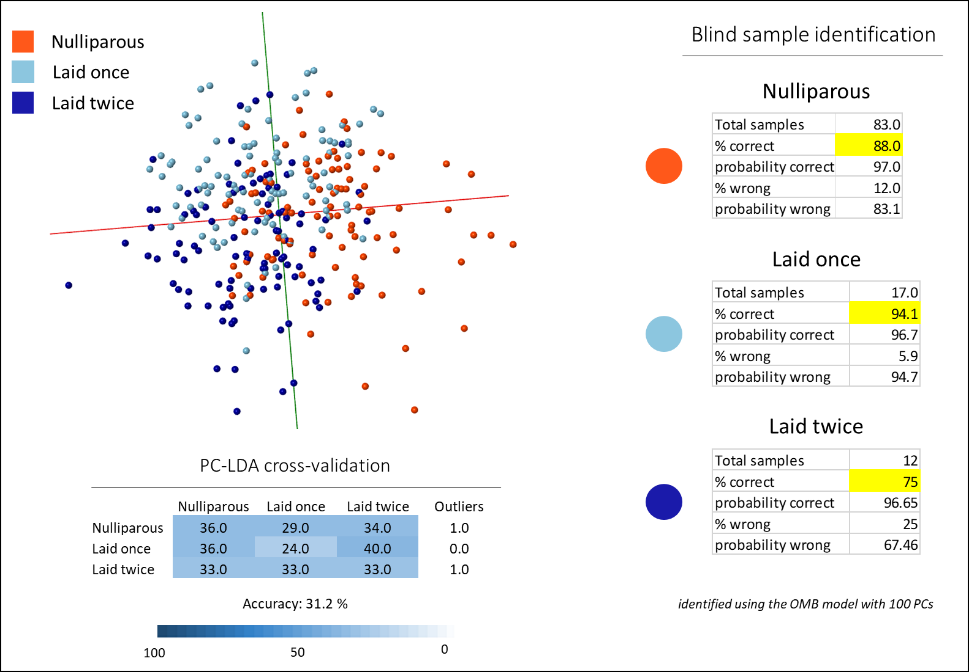
*Classifications were randomly assigned to the samples and the model with the three parity classes re-built and cross-validated. The model achieved an accuracy of only 31 %. Using the model with the correct parity information, blind samples were identified using a recognition software; between 75 and 94 % of samples per class were correctly identified.*

**Supplemental Figure 5:** ***Randomised model of the age separation model based on field collected mosquitoes and blind sample identification results.***

**
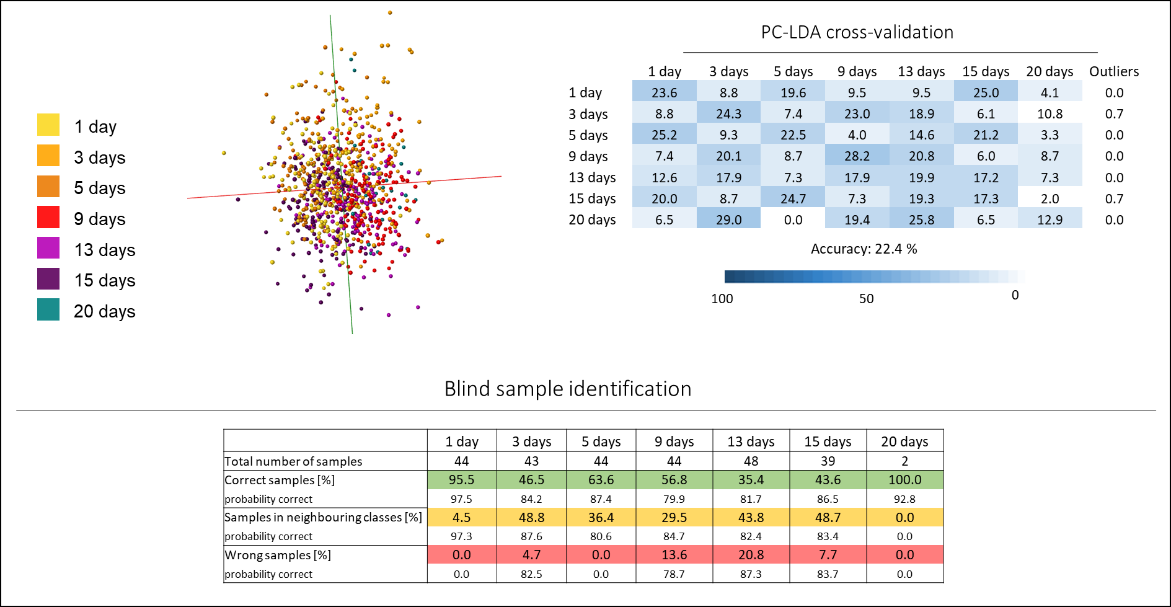
***Age classifications were randomly assigned to the samples of the combined data set and the model with seven age classes re-built and cross-validated. The model achieved an accuracy of 22 %; separation according to age was not possible. Using the model based on the correct mosquito age, blind samples were identified using a recognition software; on average 63 % of samples were correctly identified, 30 % were identified to belong to the neighbouring classes and 7 % of samples were identified wrongly.*

**Supplemental Figure 6:** ***Separation of age groups based on data collected from mosquito abdomens.***


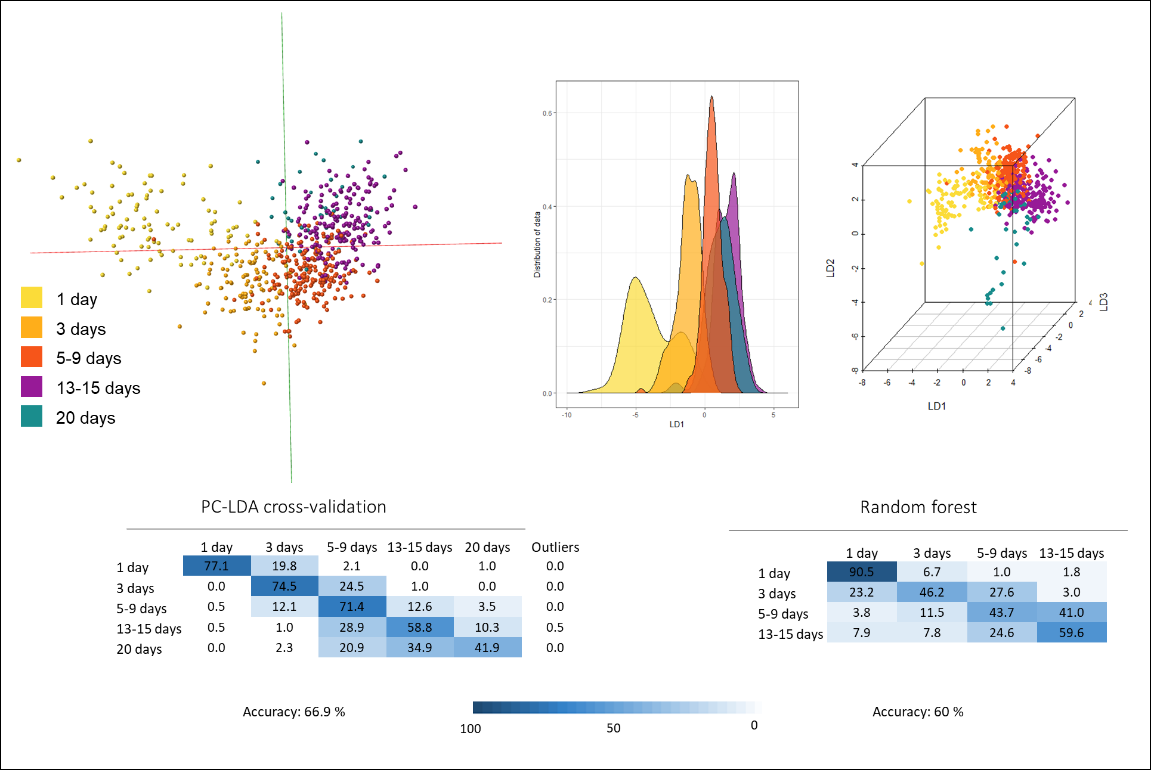
*Mosquitoes were dissected and only their abdomens were analysed with REIMS and used to build age models. The specimens were divided into five age classes and analysed via PC-LDA and random forest analysis, leading to a continuous distribution of age classes from young to old specimens. Cross-validation in OMB achieved a classification accuracy of 66.9 %, whereas random forest identified 60 % of samples correctly with a majority of the remaining samples being placed in the neighbouring classes. For random forest analysis, sample numbers are evened out across classes; due to low sample numbers, the 20-day class was removed from this analysis.*

**Supplemental Figure 7:** ***Randomised model of the age model generated based on mosquito abdomens.***


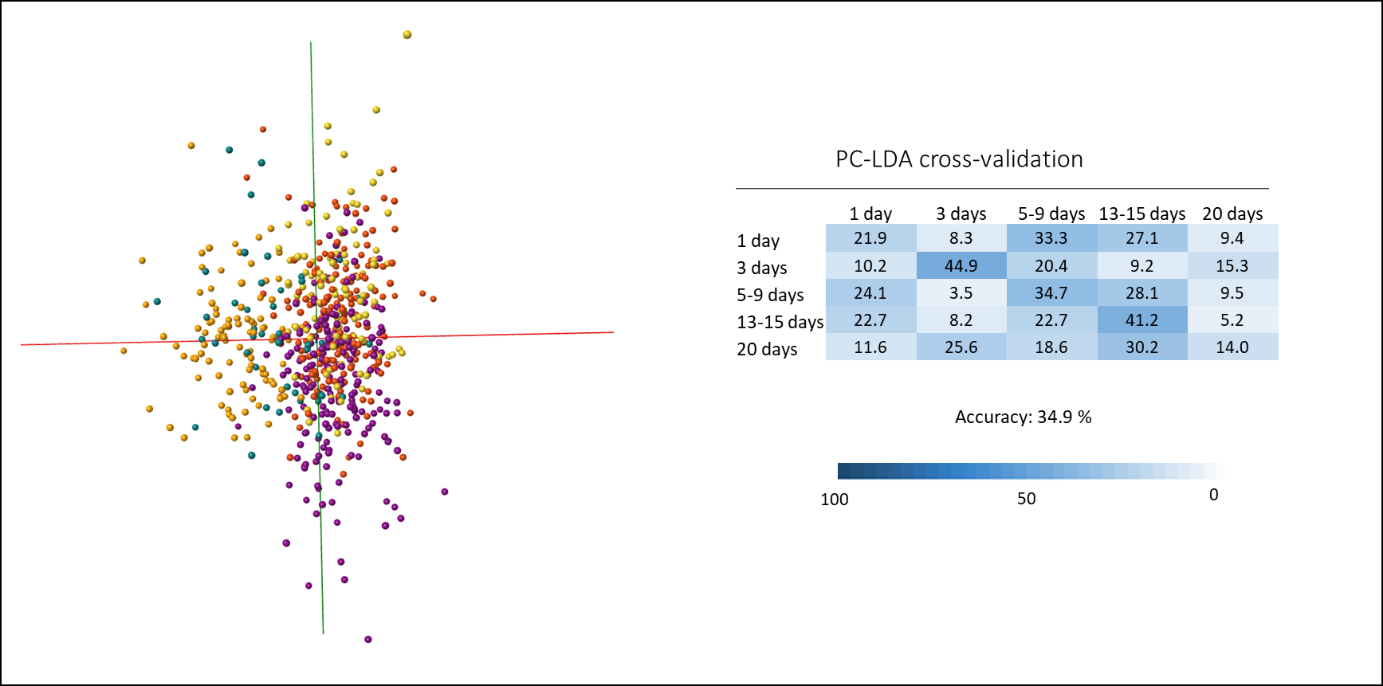
*The model based on only mosquito abdomens was re-built in OMB using randomly assigned classifications. The new model was then cross-validated, reaching an accuracy of only 35 %.*

**Supplemental Figure 8:** ***Six class model of age separation based on mosquitoes from semi-field conditions.***


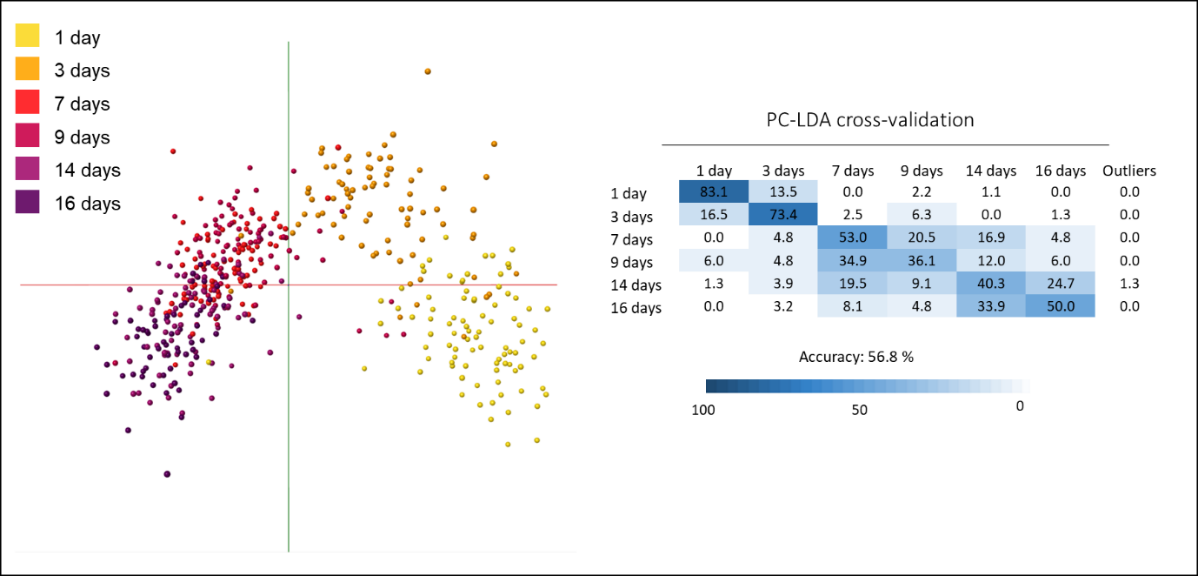
*The mosquitoes raised in the semi-field station were collected at six different time points. Here, the mosquitoes were left in their original age class without forming brackets to reduce class number. The built PC-LDA model was cross-validated and reached an accuracy rate of 56.8 %.*

**Supplemental Figure 9:** ***Randomised model of the three-class age separation based on mosquitoes from semi-field conditions***


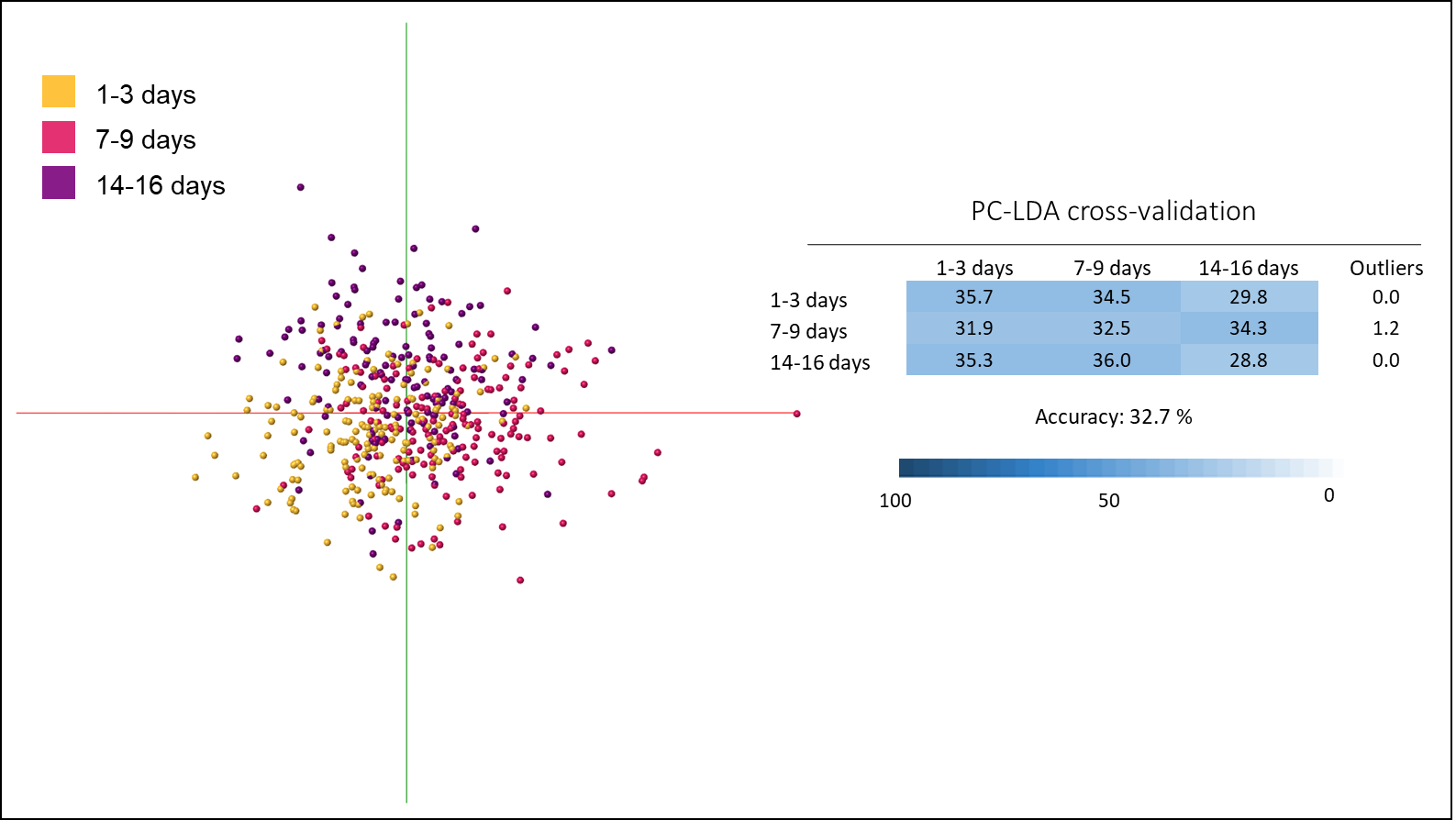
*The model based on semi-field raised mosquitoes was re-built using randomly assigned age brackets and cross-validated in OMB, reaching an accuracy of 32.7 %.*

**Supplemental Figure 10:** ***Separation of wild mosquitoes which had been collected in houses and kept in the insectary for 0, 4 and 8 days.***


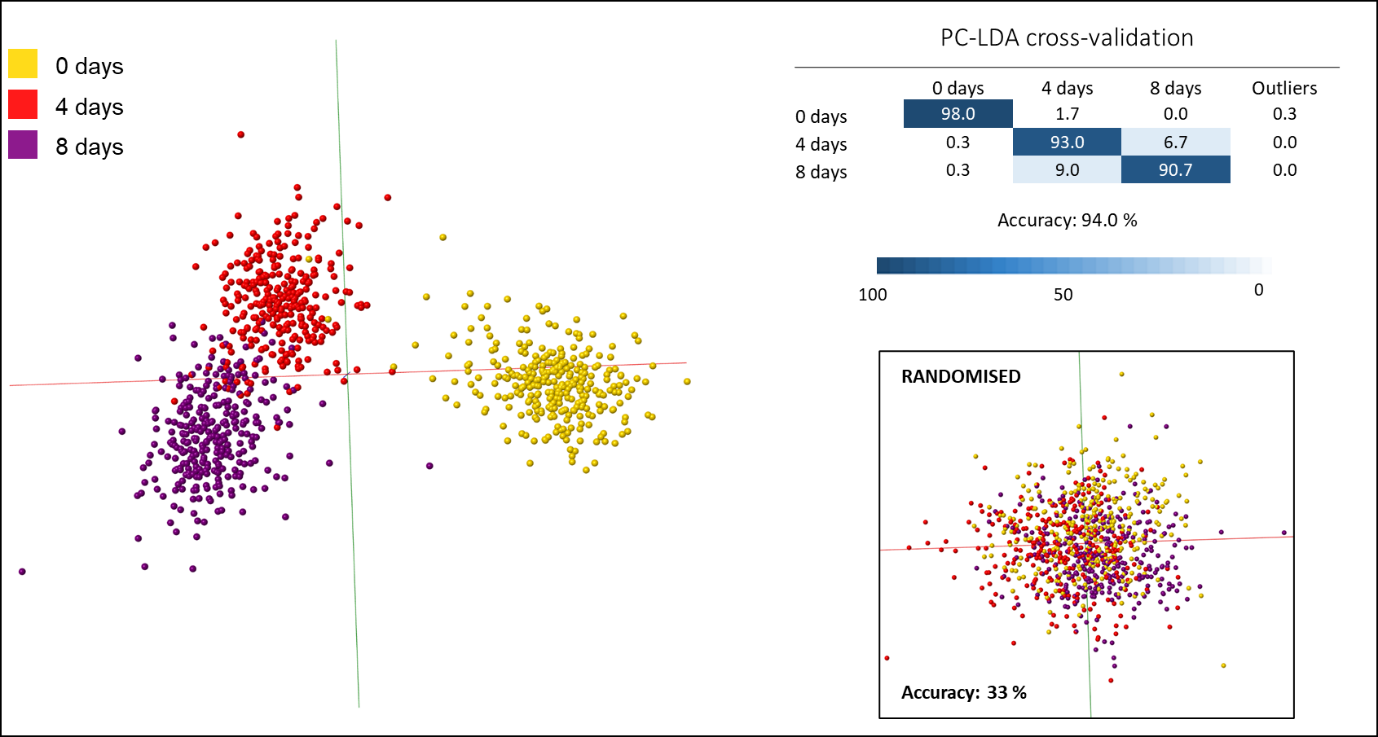
*Adult mosquitoes collected in houses were transferred to the insectary and removed after 0, 4 and 8 days. A PC-LDA model built using these three categories reached an accuracy rate of 94 % following cross-validation. A model based on randomly assigned classes had an accuracy of only 33 %.*

***Supplemental Figure 11:*** ***Separation of mosquitoes infected with* Plasmodium berghei *from non-infected specimens.***


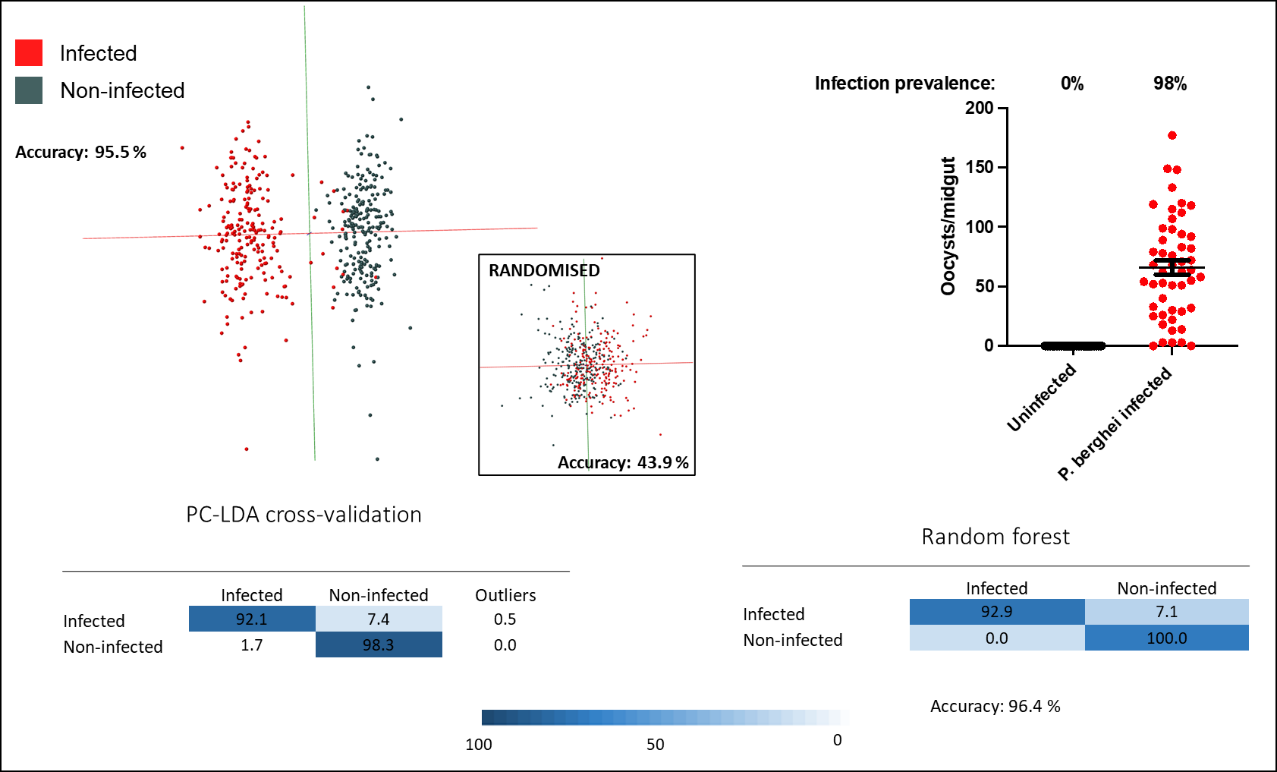
*Mosquitoes were separated into two categories after having been offered either* Plasmodium berghei *infected or uninfected blood meals. Examination of a mosquito subset revealed a infection prevalence of 98 %. Analysis of the REIMS data with PC-LDA and subsequent validation showed that 95.5 % of samples were placed into the correct categories. Random forest analysis using a 70 %/30 % data split for training and testing resulted in a accuracy rate of 96.4 %. The model was re-built using random assignment of infected and non-infected status to samples and reached an accuracy of 43.9 %.*

# **Supplementary Document 1 – Reflexivity Statement**

1. **How does this study address local research and policy priorities?**

Mosquito borne disease (malaria, and increasingly dengue) are prevalent across Burkina Faso placing an immense burden on health and well-being. Targeting the mosquito is the most effective means of preventing transmission of these diseases but assessing the impact of alternative interventions typically involves clinical trials which are very resource intensive and take several years to complete. The dynamics of malaria transmission means that the age of the mosquito population is a useful proxy for disease transmission potential. Hence tools that can estimate population age could prove useful in providing local, context specific information on the disease risk and the impact of alternative interventions at a fraction of the cost of current gold standard methods.

1. **How were researchers from Burkina Faso involved in study design?**

Researchers from two internationally renowned institutes in Burkina Faso made substantial contributions to study design. Researchers at the Institut de Recherche en Sciences de la Santé (IRSS) provided access to, and expertise on the use of, semi-field stations; IRSS are co-leads in complementary studies using alternative technologies to assess mosquito age and generously shared their learnings from this work. The Centre National de Recherche et de Formation sur le Paludisme (CNRFP) and the Liverpool School of Tropical Medicine (LSTM) have had a collaborative research partnership since 2008, with multiple joint research grants and publications. AS and MG from CNFRP were part of the management team for the current study and played a major role in designing and implementing the study.

1. **How has funding been used to support the local research team?**

This project was directly funded from two sources and 22 % of the total budget was sent directly to CNRFP (a proportion of which was used to support IRSS refurbishment of the semi-field stations). The project also leveraged additional funds from an UKRI Future Leaders Fellowship to ESS which were transferred to CNRFP to support mosquito collections.

1. **How are research staff who conducted data collection acknowledged?**

All colleagues who contributed directly to the project are included as authors. The technical team that supported mosquito rearing in Burkina Faso (and LSTM) are included in the acknowledgements.

1. **Do all members of the research partnership have access to study data?**

Data from the study has been uploaded onto a repository and so is available for all to access. A dropbox folder was used throughout the study for data management and all members of the management team had access to this.

1. **How was data used to develop analytical skills within the partnership?**

As this was a multidisciplinary project, involving experts in proteomics and entomologists, it was not practical to upskill all authors in all analytical methods. One author (IW) was solely responsible for the model building, members of the core team (IW, AS, MG, ESS and HR) met regularly (approximately every 2 months) to discuss the interpretation of data from the models.

1. **How have research partners collaborated in interpreting study data?**

See above.

1. **How were research partners supported to develop writing skills?**

The lead and corresponding authors (IW and HR) drafted the manuscript with substantial input from AS, MG and ESS.

1. **How will research products be shared to address local needs?**

The research is still at quite an early stage in the translational pathway. Whilst we have demonstrated that the REIMS technology can theoretically be used to estimate population ages of wild caught mosquitoes, the methodology used in the current study utilised a high end, non-portable mass spectrometer. The next phase of the project would involve a technology transfer aspect to adapt the methodology to a field-applicable portable instrument.

1. **How is the leadership, contribution and ownership of this work by LMIC researchers recognised within the authorship?**

Nine of the 16 authors are based in Burkina Faso although, in this instance both first and last authors are in the UK. We have reflected carefully on this and considered shared first authorship but the consensus was that the vast majority of the work was led and conducted by IW (who was employed full time on the project) and, whilst all authors made important contributions, shared first authorship was not appropriate in this instance. The project was conceived, funded and led by authors based in Liverpool.

1. **How have early career researchers across the partnership been included within the authorship team?**

Nine of the 16 authors identify as early career researchers (IW, AS, JW, JH, RS, SI, MT, ZS, & EB). Two of these (IW and AS) were part of the management team.

1. **How has gender balance been addressed within the authorship?**

Eleven authors are male five authors female (including 1^st^ and senior author).

1. **How has the project contributed to training of LMIC researchers?**

The project contributed to reinforce the analytical capacities of the two LMIC teams involved in the project in terms of study design, data analyses and how to use predictive models to validate age grading; data management skills were also enhanced as part of the project.

1. **How has the project contributed to improvements in local infrastructure?**

This project led to the refurbishment of semi-field stations at IRSS.

1. **What safeguarding procedures were used to protect local study participants and researchers?**

The study did not directly involve children or vulnerable adults. However mosquitoes were collected from houses in the study sites. Whilst the PI of the study had received safeguarding training, on further reflection, this should have been rolled out to all involved in these collections. Lessons have been learnt for future studies.
